# Subgroup-aware suicide risk prediction via functionally adaptive interaction regularized regression

**DOI:** 10.1038/s44184-026-00229-y

**Published:** 2026-07-22

**Authors:** Sharmin Afrose, William H. Kazanis, Jodie Trafton, Anuj Kapadia, Alina Peluso

**Affiliations:** 1 Oak Ridge National Laboratory, Oak Ridge, TN USA; 2 Veterans Health Administration, Palo Alto, CA USA

**Keywords:** Health care, Medical research, Psychology, Psychology, Risk factors

## Abstract

Suicide is a major public health issue, and machine learning offers promising tools to identify individuals at heightened risk. However, conventional models often fail to optimize performance across population subgroups, which can lead to inconsistent detection and outcomes. Using 2013–2023 data from the U.S. National Survey on Drug Use and Health (NSDUH), this study introduces the Functionally Adaptive Interaction Regularization-Penalized Logistic Regression (FAIR-PLR) framework: a logistic extension of the linear FAIR framework that combines group-covariate interaction terms with subgroup-size-weighted elastic-net regularization. Benchmarked against standard logistic regression, pooled elastic net with uniform weights, a decision tree, and subgroup-specific PLR across single subgroups (age, sex, race, BMI, insurance, rurality) and cross-sectional strata of psychological distress and treatment, FAIR-PLR matches or improves upon every baseline in overall performance while narrowing subgroup-level gaps in AUC, true positive rate, and precision at top-risk thresholds. FAIR-PLR gives a subgroup-balanced tool for national-scale suicide risk stratification.

## Introduction

Suicide remains a leading cause of preventable death worldwide and represents a significant public health concern^1,2^. Globally, more than 720,000 people die due to suicide every year^3^. In the United States alone, over 49,316 individuals died by suicide in 2023^4^, with millions more experiencing suicidal thoughts or engaging in non-fatal attempts.

Machine learning (ML) techniques are increasingly proposed to enhance risk assessment by modeling complex patterns in clinical records, surveys, or social media data^5–9^. Such approaches have also been implemented in large health systems to proactively direct clinical attention to patients at elevated suicide risk^10^. However, despite promising overall performance of ML models, many ML models overlook maximizing subgroup performance and lack subgroup-level interpretability in high-risk suicide prediction^11–13^. Understanding these subgroup differences is critical, as uneven model performance can mask elevated risk in specific demographic or socioeconomic groups, leading to under-detection and potentially widening existing health disparities. Many recent studies indicate that subgroup performance differences often arise when a single “one-model-fits-all” approach is applied across different groups of populations. For example, Coley et al.^11^ reported that logistic regression and random forest models predicted suicide risk with strong overall accuracy but had substantially weaker performance for Black and American Indian/Alaska Native patients compared with White, Hispanic, and Asian patients, highlighting gaps in subgroup performance reliability. Similarly, a recent systematic review and meta-analysis of 54 machine learning models for suicidal ideation, attempts, and deaths found pooled AUCs around 0.86, but with wide variability in sensitivity depending on the population and modeling approach^14^. Algorithmic bias has been documented across clinical ML applications beyond suicide prediction, including breast-cancer pathology classification, where subgroup performance gaps persisted after standard subgroup-performance audits^15^. Recent ML-for-health surveys^16^ flag equitable subgroup performance as an open problem, arguing that inclusive model design has both ethical and clinical-utility consequences^17^. Subgroup performance should therefore be a training-time objective, not a post-hoc audit.

The NSDUH data^18^, from the Substance Abuse and Mental Health Services Administration (SAMHSA)^19^, provides nationally representative data on mental health, substance use, and demographics, and is frequently used to study suicidal thoughts and behaviors^20–25^. Many NSDUH studies, however, are cross-sectional (i.e., data collected within a single year) and descriptive (i.e., providing prevalence estimates or associations rather than predictive modeling). For instance, logistic-regression analyses of the 2019 NSDUH found that 4.9% of adults reported suicidal thoughts, 1.37% reported plans, and 0.56% reported attempts^21^. Younger adults (18–25 years), individuals with alcohol or illicit-drug dependence, and those with major depressive episodes had substantially higher odds of suicidal ideation and attempts^21^. Similarly, trends from the 2009-2020 NSDUH data show that suicidal ideation among adults with opioid-use disorder rose from 22.8% to 29.8%, associated with younger age, non-metropolitan residence, co-occurring depression, and alcohol or sedative use disorders^22^. Another study of 2015–2019 respondents found that past-month binge drinking or prescription drug misuse was more strongly associated with suicidal ideation and attempts among adults over 25 years than among college-aged adults^23^. While these analyses highlight important correlates, they rarely rank individuals by predicted risk or evaluate subgroup-level predictive performance. Assessing predictive accuracy across subgroups is essential for identifying systematic gaps in detection, ensuring that high-risk individuals are not obscured by aggregate trends, and guiding the development of more reliable, interpretable models for population-level suicide-risk assessment.

Most existing suicide-risk prediction models prioritize high overall accuracy while undermining differences across demographic or clinical subgroups and offering limited interpretability. Similarly, many prior NSDUH-based studies often analyze single survey years and rely on a narrow set of predictors. To address these limitations, we leverage a decade of harmonized NSDUH data (2013–2023) to build interpretable, subgroup-robust models using the FAIR-PLR framework.

Several prior research directions shape this work. Subgroup-aware interpretable ML has been approached through rule-based learners such as RIPPER and SkopeRules, Explainable Boosting Machines, and generalized additive models, which keep coefficient-level transparency while allowing local adaptation^11–13^. Post-hoc explanation tools like SHAP and LIME help interpret opaque models but do not guarantee equitable cross-subgroup performance; that balance must come from the training objective. In the penalized-regression literature, Zhang et al.^26,27^ showed that changes to the logistic log-likelihood, including cost-sensitive reweighting and variable discretization, improve performance on highly imbalanced data, which matters for suicide prediction because cases are a small fraction of the sample. Decision-support frameworks that combine predictive modeling with multi-criteria evaluation or transfer learning have been proposed in other public-health contexts^28,29^ and suggest how a subgroup-balanced predictor can plug into a broader policy workflow.

This work builds directly on the FAIR framework of Smolyak et al.^30^, which showed that a full linear-interaction model between group indicators and covariates, combined with group-size sample weighting and independent per-group penalties, can improve predictive performance on imbalanced demographic subgroups without sacrificing linear-model interpretability. The original FAIR framework was formulated for continuous outcomes under ordinary least squares. We extend it in four ways. First, we replace the squared-error loss with the binomial deviance loss so the framework applies to binary suicidal outcomes; the result is FAIR-PLR (Functionally Adaptive Interaction Regularization–Penalized Logistic Regression). Second, we replace the per-group penalties with a single shared elastic-net penalty whose strength is chosen by cross-validation, giving one coefficient path and simpler tuning. Third, we apply the framework to a decade-scale harmonized national survey (NSDUH, 2013–2023, 452,344 adults, 33 predictors) rather than the smaller tabular datasets of the original work. Fourth, we incorporate NSDUH’s person-level survey weight directly into the training loss, yielding a single combined weight (Methods, Eq. (4)) that equalizes subgroup loss contributions while preserving within-subgroup national representativeness; this makes the fitted model nationally representative rather than sample-representative. The result is one interpretable, subgroup-balanced model for national-scale suicide-risk stratification.

Beyond single-variable associations, our study investigates how combinations of factors jointly shape suicide-related outcomes through cross-sectional subgroup interactions. We focus on how serious psychological distress (SPD) interacts with key demographic and contextual characteristics. For example, sex × SPD and age × SPD interactions reveal that distress may exert stronger effects among females and younger adults, respectively. Marital status × SPD interactions capture the protective influence of marriage and elevated risk among unmarried or separated individuals. Rurality × access-to-care (insurance coverage or treatment receipt) highlights how limited mental health and substance use treatment availability in nonmetropolitan areas may exacerbate vulnerability. Finally, BMI × SPD interactions suggest that psychological distress may elevate suicide risk even among individuals with a healthy body weight.

Using eleven years of NSDUH data, we harmonized 33 variables encompassing demographics, substance use, treatment receipt, and mental health indicators through a custom code-harmonization dictionary. FAIR-PLR extends the FAIR framework by applying elastic-net logistic regression with group-covariate interaction terms weighted by subgroup size. These terms yield subgroup-specific coefficients, allowing interpretable comparisons across demographic and psychological strata such as age, sex, marital status, urbanicity, and BMI. For benchmarking, we train separate penalized logistic regression (PLR) models independently within each subgroup. Model performance is evaluated using AUC-ROC and true-positive rates among the top 1% and 5% of predicted risk.

The contributions of this study are as follows: (1) Integration of eleven years (2013-2023) of harmonized NSDUH data encompassing 33 key predictors of suicide risk. (2) Adaptation of the FAIR framework to penalized logistic regression, introducing FAIR-PLR: a logistic extension of Smolyak et al.’s linear-regression FAIR that uses a single shared elastic-net penalty with subgroup-size-weighted observations to balance performance across diverse populations on binary suicidal outcomes. (3) Demonstration that FAIR-PLR matches or exceeds separate PLR models in overall performance while substantially reducing subgroup disparities. (4) Characterization of how multiple demographic, socioeconomic, and psychological factors interact to shape suicide risk.

Overall, this integrative, cross-sectional analysis advances suicide-risk prediction by enhancing interpretability, reducing subgroup performance gaps, and providing data-driven insights to inform targeted public-health interventions.

## Methods

**Fig. 1:**
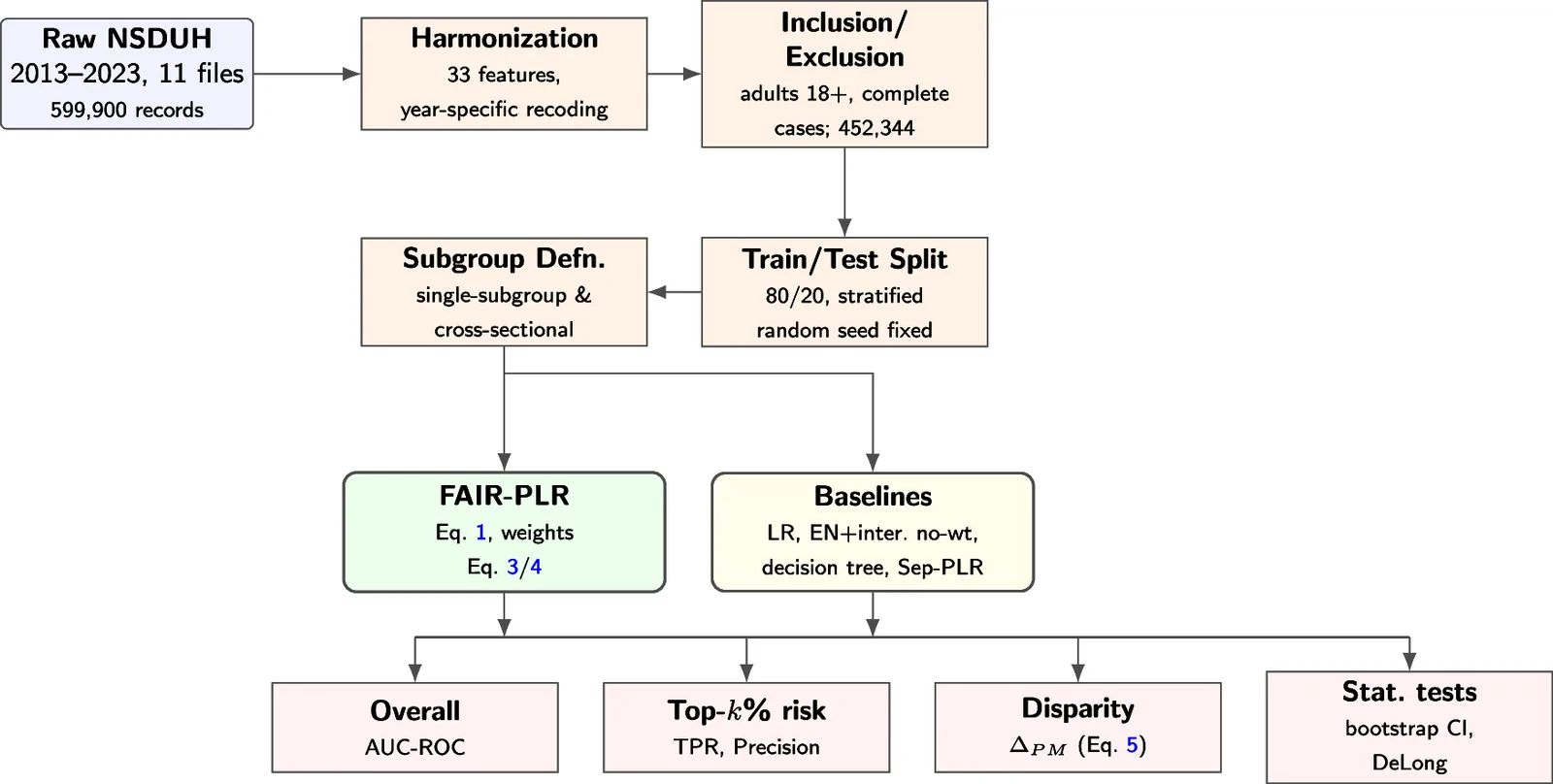
End-to-end study pipeline for developing and evaluating FAIR-PLR on harmonized NSDUH data (2013–2023), spanning data harmonization, cohort filtering, subgroup definition, model fitting, and subgroup-stratified performance evaluation. Raw NSDUH public-use files are harmonized into a constant 33-feature schema, filtered to adult respondents with complete key variables, split 80/20 into train/test sets, and stratified along two subgroup axes: single-subgroup (age, sex, race, BMI, insurance, rurality) and cross-sectional (demographic ×SPD or treatment). FAIR-PLR is trained with interaction-augmented features and subgroup-size-weighted elastic-net regularization, alongside four baseline models (standard logistic regression, pooled elastic net with interactions but uniform weights, decision tree, and subgroup-specific PLR). All models are evaluated on the same held-out test set for overall AUC-ROC, top-k% true-positive rate (TPR) and precision, subgroup disparity Δ_*PM*_, and bootstrap/DeLong statistical significance tests. NSDUH National Survey on Drug Use and Health, BMI body mass index, SPD serious psychological distress, FAIR-PLR functionally adaptive interaction regularization-penalized logistic regression, PLR penalized logistic regression, AUC-ROC area under the receiver operating characteristic curve, TPR true positive rate, Δ_*PM*_ performance-metric disparity ratio.

Figure 1 gives a schematic overview of the study pipeline, from raw NSDUH public-use files through harmonization, subgroup definition, FAIR-PLR and baseline model fitting, and subgroup-stratified evaluation. Each stage is detailed in the subsections below.

### Data Source

The National Survey on Drug Use and Health (NSDUH)^18^ is an annual survey sponsored by the Substance Abuse and Mental Health Services Administration (SAMHSA)^31^. NSDUH is one of the primary sources of nationally representative data on substance use and mental health among the United States population aged 12 and older. NSDUH collects detailed information on demographics, substance use disorders, mental health topics, and treatment received for substance use or mental-health problems. NSDUH fits this study on three counts. It samples the civilian, non-institutionalized U.S. population at national scale, with annual releases that keep core domains consistent across more than a decade. It carries suicidal ideation, plans, and attempts alongside psychological distress, substance use, and demographics, covering the main correlates of suicide risk. And its public-use files allow reproducible secondary analyses. The target population is civilian, non-institutionalized U.S. residents aged 12 and older. By design, the NSDUH sampling frame excludes unsheltered homeless individuals, incarcerated populations, and active-duty military personnel, which bounds the external validity of any NSDUH-based prediction model.

### Data Harmonization and Preprocessing

For this study, we use the delimited public-use NSDUH files for the 2013–2023 survey years. The dataset requires extensive cleaning because variable coding and structure vary across years. We extract 33 key features associated with suicide across the years. Supplementary Table 1 summarizes how we harmonize year-specific variables to a constant set of definitions. The key outcome variable is adult thought about, planned, or attempted suicide in the past year (ADSUITPAYR). Health-insurance coverage is derived by consolidating multiple insurance type variables into a single categorical variable with five levels: private insurance, Medicaid/CHIP, Medicare, other insurance, and no insurance. The year-specific cleaning process ensured consistent variable definitions and coding schemes across the 11-year dataset by preserving the integrity of the original survey responses.

Supplementary Fig. 1 shows the data-filtering process for the 2013–2023 NSDUH dataset. The combined initial data has 599,900 records. After excluding 3550 cases with missing poverty information, 596,350 records remained. We then remove 142,511 individuals under the age of 18 to restrict the sample to adults, leaving 453,839 records. Finally, we exclude 1495 cases with missing values in the variable indicating receipt of substance use or mental health treatment (RCVSUTOMHT), resulting in a final analytical sample of 452,344 adult records.

We additionally extract the NSDUH person-level analysis weight for each respondent, which represents the number of U.S. adults that respondent stands for. Because NSDUH revised its analysis-weight definition during the 2013–2023 period, the extracted variable depends on the survey year: ANALWT_C for 2013–2019, ANALWTQ1Q4_C for 2020 (a COVID-specific weight built from Quarters 1 and 4 only), and ANALWT2_C for 2021–2023 (revised for the addition of web interviews). We harmonize these three weights into a single column SURVEY_WEIGHT. Each annual weight is calibrated by SAMHSA to Census control totals, so dividing every respondent’s weight by the number of pooled years (*Y* = 11) places all eleven years on a single-year-equivalent population scale; this is SAMHSA’s canonical multi-year pooling convention and is applied before the weight enters the training loss.

### Descriptive summary statistics

Table 2 summarizes suicide-related outcome categories (*Y* = 0: no suicidal ideation/behavior; *Y* = 1: any suicidal ideation/behavior) using the harmonized 2013–2023 NSDUH dataset. Cross-sectional statistics capture how combinations of demographic, psychological, and health-related factors jointly influence these outcomes. Interactions such as age ×serious psychological distress (SPD), sex ×SPD, and marital status ×SPD indicate that distress has stronger associations with suicidal ideation or behavior among younger adults, females, and unmarried individuals. BMI ×SPD interactions further suggest that psychological distress elevates risk even among those with a healthy weight.

Supplementary Table 1 presents overall respondent characteristics by suicide-related outcomes. In our sample, suicidal thoughts, plans, or attempts were more common among younger adults (e.g., 53.9% in ages 18–25 vs. 2.5% in ages 65+), females, individuals who had never been married, and those with lower income. These experiences were also more prevalent among respondents reporting serious psychological distress, substance use disorders, work-related functional impairments, and self-reported feelings of tiredness or worthlessness.

### FAIR-PLR: objective function and interaction construction

We adapt the FAIR framework of Smolyak et al.^30^, originally formulated for linear regression with continuous outcomes and independent per-group penalties, to penalized logistic regression for binary suicidal outcomes under a single shared elastic-net penalty. Let ${\{({x}_{i},{y}_{i})\}}_{i = 1}^{N}$ denote the training sample where ${x}_{i}\in {{\mathbb{R}}}^{p}$ is the covariate vector and *y*_*i*_ ∈ {0, 1} is the binary outcome. Let *z*_*i*_ ∈ {1, …, *K*} index the FAIR subgroup (e.g., age category, sex, race, BMI, insurance, or rurality), and let *k*_0_ denote the most prevalent reference subgroup. The FAIR-PLR objective is:

1 $${\mathop{\min }\limits_{\beta }}\,\frac{1}{N}\mathop{\sum }\limits_{i=1}^{N}{w}_{i}\cdot \ell \left({y}_{i},\sigma ({\tilde{x}}_{i}^{\top }\beta )\right)\,+\,\lambda \left[\alpha \parallel \beta {\parallel }_{1}+\frac{1-\alpha }{2}\parallel \beta {\parallel }_{2}^{2}\right],$$

where *σ*( ⋅ ) is the logistic function, $\ell (y,p)=-y\log p-(1-y)\log (1-p)$ is the binomial deviance loss, and *α* ∈ [0, 1] is the elastic-net mixing parameter. The regularization strength *λ* is selected automatically by 20-fold cross-validation (R package glmnet, metric: AUC).

The augmented covariate vector ${\tilde{x}}_{i}$ encodes group–covariate interaction terms. Starting from *x*_*i*_ augmented with a bias feature (so $\dim ({x}_{i})=1+p$), we construct2where {*k*_1_,…,*k*_*K*−1_} = {1,…,*K*}⧹{*k*_0_} are the non-reference subgroups and  is the indicator function. Each observation therefore occupies a (1 + *p*)*K*-dimensional augmented feature space. Fitting a single elastic-net logistic regression on ${\tilde{x}}_{i}$ yields a stacked coefficient vector *β* = [*β*^(0)^; *β*^(1)^;…;*β*^(*K*−1)^], where *β*^(0)^ is the reference-group coefficient block and *β*^(*k*)^ is the incremental deviation for subgroup *k*. The subgroup-specific coefficient is *β*^(0)^ + *β*^(*k*)^ for non-reference subgroups and *β*^(0)^ for the reference.

The observation weight *w*_*i*_ equalizes each subgroup’s aggregate contribution to the loss while keeping within-subgroup national representativeness intact. The classical subgroup-size-only form is

3 $${w}_{i}^{\,\mathrm{size}\,}=\frac{{n}_{\min }}{{n}_{k(i)}},\qquad {n}_{k}=| \{i:{z}_{i}=k\}|,\qquad {n}_{\min }={\mathop{\min }\limits_{k}}\,{n}_{k},$$

where *n*_*k*_ is the raw count of training observations in subgroup *k*. Under (3) the smallest subgroup receives weight 1 and larger subgroups receive fractional weights in (0, 1), so every subgroup contributes the same aggregate mass ${n}_{\min }$ to the weighted loss. This form is the direct analog of Smolyak et al.^30^.

### Combined subgroup and survey weight

Equation (3) treats each respondent as representing a single person. NSDUH is a probability sample that oversamples some demographics, so a respondent’s sampling weight *s*_*i*_ records how many U.S. adults that respondent stands for. To make the fitted model nationally representative rather than sample-representative, we extend (3) to replace the raw counts *n*_*k*_ with weighted subgroup totals ${\hat{n}}_{k}={\sum }_{i\in {S}_{k}}{s}_{i}$, where *S*_*k*_ = {*i* : *z*_*i*_ = *k*}. Substituting gives

4 $${w}_{i}=\frac{{s}_{i}}{Y}\cdot \frac{{\mathop{\min}\nolimits_{k}}\,{\hat{n}}_{k}}{{\hat{n}}_{k(i)}},\qquad Y=11,$$

where the factor 1/*Y* applies SAMHSA’s canonical multi-year rescaling so that eleven pooled years of data sum to a single-year-equivalent U.S. population rather than eleven copies of it. The combined weight has two properties: (i) **cross-subgroup equalization,** ${\sum }_{i\in {S}_{k}}{w}_{i}$ is the same for every subgroup *k*, so each subgroup contributes equal aggregate mass to the loss; and (ii) **within-subgroup national representativeness**, for any *i*, *j* ∈ *S*_*k*_, *w*_*i*_/*w*_*j*_ = *s*_*i*_/*s*_*j*_, so two respondents in the same subgroup retain their original NSDUH relative representation. Equation (4) is therefore the unique (up to a global scalar) weight that enforces both properties, and it is the weight used for all main-text FAIR-PLR results.

### Baseline Models

We benchmark FAIR-PLR against four baselines, all fit on the same 80/20 stratified train/test split. Two serve as standard interpretable references; the other two are ablations that turn off specific FAIR-PLR components, so any performance gap can be attributed to a known cause:

1. **Decision tree** (CART). A sklearn.tree.DecisionTreeClassifier with max_depth tuned by 5-fold cross-validation over {3, 5, 7, 10, 15}. We include CART as a non-linear interpretable comparator: it captures threshold-style interactions that linear models miss, and its yes/no splits are easy for clinicians to read.
2. **Standard logistic regression** (pooled, no interactions, no penalty). A single sklearn.linear_model.LogisticRegression fit on the 33 harmonized features with no regularization. We include it as the simplest reference; the gap between this baseline and FAIR-PLR measures the combined value of the interaction terms and the subgroup-size weighting on the same feature set.
3. **Pooled elastic-net logistic regression**. This baseline uses the same augmented feature vector ${\tilde{x}}_{i}$ as FAIR-PLR (equation 2) with group–covariate interactions but uniform observation weights (*w*_*i*_ ≡ 1), dropping the subgroup-size weighting. We include it as the targeted ablation that turns off the subgroup-size weighting while keeping the interaction terms, so the gap to FAIR-PLR isolates the contribution of the weighting itself.
4. **Pooled subgroup elastic-net logistic regression** (Separate-PLR). A single elastic-net logistic regression fitted for each FAIR subgroup. We include it as the standard subgroup-by-subgroup alternative, which lets us test whether a single FAIR-PLR fit can match many independent per-subgroup fits without paying the cost of small per-subgroup sample sizes.

All baselines are evaluated with the same metrics (AUC-ROC, TPR, and Precision at top 1% and top 5% risk thresholds; see Performance Metrics and Evaluation) and on the same subgroup definitions.

### Subgroup definitions and interaction terms

We define subgroups along two complementary axes. *Pre-exposure subgroups* are built from variables that are either immutable or established prior to the outcome window: age, sex, race/ethnicity, BMI, health insurance, and rurality. These subgroups do not overlap with the variables used to define the outcome and are the primary surface on which subgroup-performance claims are evaluated. *Cross-sectional subgroups* pair a demographic or health variable with a clinical variable that is itself a predictor: age ×serious psychological distress (SPD), sex ×SPD, marital status ×SPD, BMI ×SPD, and rurality ×treatment receipt. These cross-sectional strata are a clinical lens for examining effect heterogeneity across risk-relevant strata. SPD and treatment status remain features of the FAIR-PLR model, and the cross-sectional analysis assesses coefficient heterogeneity rather than generalization to unseen SPD status. For each subgroup axis, the reference subgroup *k*_0_ in the interaction expansion 2 is chosen as the most prevalent level (e.g., Female for sex, 18–25 years old for age, Non-Hispanic White for race, Healthy for BMI, Private plan for insurance, Large Metropolitan for rurality).

### Hyperparameter selection

FAIR-PLR has two hyperparameters. The elastic-net mixing parameter *α* is fixed across all main results at *α* = 0.5 (balanced *L*_1_/*L*_2_). The regularization strength *λ* is selected by 20-fold cross-validation within glmnet, using AUC as the selection criterion. For the baseline models, the elastic-net regularization strength is tuned over the same grid as the original agnostic-PLR baseline (*C* ∈ {10^−3^, 10^−2^, 10^−1^, 1, 10, 10^2^}) via 5-fold cross-validation on the training set.

### Performance metrics and evaluation

We evaluate models by ranking individuals according to the predicted probability of suicidal thoughts, plans, or attempts and assess performance using three complementary metrics: AUC-ROC for the overall dataset, True Positive Rate (TPR) for the top 1% and top 5% risk strata, and Precision for the same top 1% and top 5% risk strata. Area Under the ROC Curve (AUC-ROC) measures the model’s ability to distinguish between cases and non-cases. True Positive Rate at the top *k*% is computed as the fraction of all true positives captured within the top *k*% of predicted risk, i.e. TPR_*k*%_ = (actual positives in top*k*%)/(total actual positives); Precision at the top *k*% is the fraction of the top-*k*% subset that is truly positive, i.e. Precision_*k*%_ = (actual positives in top*k*%)/(size of top*k*% subset). TPR and Precision answer complementary questions: TPR quantifies how many of the truly at-risk individuals the model captures at the high-risk threshold, and Precision quantifies how many of the flagged individuals are truly at-risk. Both are reported for overall and subgroup-stratified evaluations.

To assess how model performance varies across demographic subgroups, we calculate a disparity metric. Let *A* be a subgroup variable with possible categories {*a*_1_, *a*_2_,…,*a*_*n*_}. For any performance metric *PM* (AUC-ROC, TPR at top 1%, TPR at top 5%, Precision at top 1%, Precision at top 5%), we define the difference in performance:

5 $${\Delta }_{PM}(A)=\frac{{\mathop{\max }\nolimits_{a}}\,\,PM(X:A(X)=a)}{{\mathop{\min }\nolimits_{a}}\,\,PM(X:A(X)=a)}$$

Where *PM*(*X*: *A*(*X*) = *a*) is the performance metric computed over test observations in class *A* = *a*. A value of Δ_*PM*_ = 1 signifies no disparity, whereas values greater than 1 reflect increasing differences between groups. Lower values of Δ_*PM*_ are therefore more desirable because they indicate that the model’s performance is consistent across subgroups.

### Statistical significance testing

To assess whether model-to-model and subgroup-to-subgroup performance differences are statistically meaningful, we compute 95% confidence intervals for AUC-ROC, TPR, and Precision via stratified bootstrap resampling with 1000 iterations on the held-out test set. For each model and each subgroup, the resampling is stratified on the outcome to preserve class prevalence. Pairwise AUC comparisons between FAIR-PLR and each baseline are performed with DeLong’s test on the same test-set predictions, reported as two-sided *p*-values. Subgroup-level differences in disparity metrics (Δ_*PM*_) are reported with bootstrap CIs under the same stratified resampling procedure. We report point estimates with 95% CIs in the main results tables and flag pairwise comparisons at *p* < 0.05 without multiple-comparison correction, noting this as a descriptive rather than confirmatory analysis.

## Results

### Descriptive findings

Table 1 details subgroup-level characteristics among adults in the NSDUH sample. Suicidal thoughts, plans, or attempts were most frequent among younger, unmarried adults with mental health or substance use disorders. Adults aged 18–25 years reported the highest prevalence (53.9%) compared with 2.5% among adults aged 65 years or older. Women more often reported suicidal experiences than men (59.0% vs 41.0%). Marital status showed strong associations: 66.6% of individuals reporting suicidal behavior had never been married, while 21.1% were married. Among those with suicidal behavior, 73.9% experienced SPD in the past year, 53.6% had a major depressive episode, 57.8% met criteria for alcohol use disorder, and 61.6% for cocaine use disorder. Many also endorsed symptoms such as persistent tiredness, low energy, or difficulty working.

**Table 1:** Summary statistics of suicidal thoughts, plans, and attempts among adults based on National Survey on Drug Use and Health (NSDUH) Data (2013-2023)

	No suicidal		Any suicidal		Total
	Ideation/Behavior		Ideation/Behavior		
	*N*	%	*N*	%	
	423,464	93.62%	28,880	6.38%	452,344
Year					
2013	34,519	8.15%	2080	7.20%	36,599
2014	38,886	9.18%	2049	7.09%	40,935
2015	40,539	9.57%	2360	8.17%	42,899
2016	39,519	9.33%	2348	8.13%	41,867
2017	| 39,328	9.29%	2530	8.76%	41,858
2018	39,593	9.35%	2692	9.32%	42,285
2019	38,859	9.18%	3008	10.42%	41,867
2020	24,293	5.74%	1791	6.20%	26,084
2021	42,600	10.06%	3152	10.91%	45,752
2022	43,513	10.28%	3563	12.34%	47,076
2023	41,815	9.87%	3307	11.45%	45,122
Age Categories					
18-25 Years Old	130,197	30.75%	15,558	53.87%	145,755
26-34 Years Old	85,378	20.16%	5657	19.59%	91,035
35-49 Years Old	113,985	26.92%	5215	18.06%	119,200
50-64 Years Old	51,491	12.16%	1728	5.98%	53,219
65 or Older	42,413	10.02%	722	2.50%	43,135
Sex					
Male	194,939	46.03%	11,846	41.02%	206,785
Female	228,525	53.97%	17,034	58.98%	245,559
Race/Hispanicity					
Non Hispanic White	257,313	60.76%	18,029	62.43%	275,342
Non Hispanic Black/African American	51,123	12.07%	2849	9.86%	53,972
Non Hispanic Native Am/AK Native	5730	1.35%	516	1.79%	6246
Non Hispanic Native HI/Other Pac Isl	2076	0.49%	125	0.43%	2201
Non Hispanic Asian	21,208	5.01%	1040	3.60%	22,248
Non Hispanic more than one race	13,830	3.27%	1743	6.04%	15,573
Hispanic	72,184	17.05%	4578	15.85%	76,762
Marital Status					
Married	180,957	42.73%	6109	21.15%	187,066
Widowed	13,202	3.12%	403	1.40%	13,605
Divorced or Separated	44,603	10.53%	3130	10.84%	47,7
Never Been Married	184,702	43.62%	19,238	66.61%	203,940
Education Categories					
Less than high school	51,062	12.06%	3702	12.82%	54,764
High school graduate	111,556	26.34%	8316	28.80%	119,872
Some college/Associate Degree	130,541	30.83%	10,965	37.97%	141,506
College graduate	130,305	30.77%	5897	20.42%	136,202
Poverty Level (% of US Census Poverty Threshold)					
Living in Poverty	75,624	17.86%	7098	24.58%	82,722
Income Up to 2X Federal Poverty Threshold	86,341	20.39%	7159	24.79%	93,500
Income More Than 2X Federal Poverty Threshold	261,499	61.75%	14,623	50.63%	276,122
Employment Status					
Unemployed	24,719	5.84%	2917	10.10%	27,636
Employed full time	219,699	51.88%	12,282	42.53%	231,981
Employed part time	64,740	15.29%	5983	20.72%	70,723
Other	114,306	26.99%	7698	26.66%	122,004
Total Family Income					
Less than $20,000	77,898	18.40%	7760	26.87%	85,658
$20,000 - $49,999	126,321	29.83%	9598	33.23%	135,919
$50,000 - $74,999	66,384	15.68%	4082	14.13%	70,466
$75,000 or More	152,861	36.10%	7440	25.76%	160,301
Health Insurance					
Medicaid/CHIP	68,802	16.25%	6998	24.23%	75,800
Medicare	16,403	3.87%	572	1.98%	16,975
Other	20,126	4.75%	1651	5.72%	21,777
Private plan	267,268	63.11%	15,487	53.63%	282,755
Uninsured	50,865	12.01%	4172	14.45%	55,037
County Metro/Nonmetro Status (Rurality)					
Large Metropolitan	191,693	45.27%	11,995	41.53%	203,688
Nonmetropolitan	78,720	18.59%	5608	19.42%	84,328
Small Metropolitan	153,051	36.14%	11,277	39.05%	164,328
Body Mass Index (BMI)					
Underweight	9944	2.35%	995	3.45%	10,939
Healthy	138,891	32.80%	9656	33.43%	148,547
Overweight	124,532	29.41%	6955	24.08%	131,487
Obesity	107,028	25.27%	7644	26.47%	114,672
Severe Obesity	23,267	5.49%	2417	8.37%	25,684
Unknown	19,802	4.68%	1213	4.20%	21,015
Drug or Alcohol Use Disorder (any past year)	231,367	54.64%	19,877	68.83%	251,244
Serious Pscyhological Distress Indicator (any past month)	25,027	5.91%	14,466	50.09%	39,493
Serious Pscyhological Distress Indicator (any past year)	54,121	12.78%	21,342	73.90%	75,463
Received Substance Use Treatment or Mental Health Treatment (any past year)	71,955	16.99%	14,949	51.76%	86,904
Major Depressive Episode (any past year)	27,898	6.59%	15,491	53.64%	43,389
Alcohol Use Disorder (any past year)	215,461	50.88%	16,708	57.85%	232,169
Marijuana Use Disorder (any past year)	63,496	14.99%	10,224	35.40%	73,720
Used Stimulant Without Own RX (any past year)	7537	1.78%	1564	5.42%	9101
Used Sedative Without Own RX (any past year)	1029	0.24%	306	1.06%	1335
Cocaine Use Disorder (any past year)	7505	1.77%	1708	5.91%	9213
Heroin Use Disorder (any past year)	1473	0.35%	434	1.50%	1907
Hallucinogen Use Disorder (any past year)	7516	1.77%	1766	6.11%	9282
Inhalant Use Disorder (any past year)	1687	0.40%	508	1.76%	2195
Oxycontin Use (any past year)	10,651	2.52%	1807	6.26%	12,458
Drug Use Disorder (any past year)	76,959	18.17%	12,574	43.54%	89,533
Binge Alcohol Use (any past month)	121,616	28.72%	10,363	35.88%	131,979
Heavy Alcohol Use (any past month)	31,410	7.42%	3261	11.29%	34,671
Difficulty in Work Response for One Month (any past year)	134,254	31.70%	22,436	77.69%	156,690
Felt Tired/Low Energy (nearly every day)	59,419	14.03%	18,353	63.55%	77,772
Felt Worthless (nearly every day)	33,265	7.86%	15,619	54.08%	48,884

Table 2 summarizes variations in suicidal thoughts, plans, and attempts across demographic, behavioral, and contextual subgroups when combined with serious psychological distress (SPD) or treatment status. These cross-sectional comparisons illustrate how multiple factors jointly shape suicide risk. Younger adults aged 18–25 years with SPD accounted for 42.2% of all suicidal cases, and females with SPD represented 46.1%. Individuals who had never been married and reported SPD comprised 51.0% of suicidal cases. Among those residing in small metropolitan areas, 20.9% of individuals with suicidal behavior had received mental health or substance use treatment. Across BMI categories, suicidal behavior was most prevalent among adults with a healthy BMI who also experienced SPD, underscoring the interplay between psychological distress and overall health context.

**Table 2:** Summary statistics of suicidal thoughts, plans, and attempts by cross-sectional variables using National Survey on Drug Use and Health (NSDUH) Data (2013-2023)

	No suicidal ideation/behavior		Any suicidal ideation/behavior		Total
	*N*	%	*N*	%	
	423,464	93.62%	28,880	6.38%	452,344
Age ×Serious Psycological Distress					
18-25 Years Old : Yes	24,734	5.84%	12,195	42.23%	36,929
26-34 Years Old : Yes	12,594	2.97%	4239	14.68%	16,833
35-49 Years Old : Yes	11,925	2.82%	3626	12.56%	15,551
50-64 Years Old : Yes	3479	0.82%	1035	3.58%	4514
65 or Older:Yes	1389	0.33%	247	0.86%	1636
18-25 Years Old : No	105,463	24.90%	3363	11.64%	108,826
26-34 Years Old: No	72,784	17.19%	1418	4.91%	74,202
35-49 Years Old: No	102,060	24.10%	1589	5.50%	103,649
50-64 Years Old: No	48,012	11.34%	693	2.40%	48,705
65 or Older: No	41,024	9.69%	475	1.64%	41,499
Sex ×Serious Psycological Distress					
Male: Yes	18,719	4.42%	8037	27.83%	26,756
Female: Yes	35,402	8.36%	13,305	46.07%	48,707
Male: No	176,220	41.61%	3809	13.19%	180,029
Female: No	193,123	45.61%	3729	12.91%	196,852
Marital Status ×Serious Psychological Distress					
Married: Yes	14,480	3.42%	4020	13.92%	18,500
Widowed: Yes	1082	0.26%	228	0.79%	1310
Divorced or Separated: Yes	6192	1.46%	2293	7.94%	8485
Never Been Married: Yes	32,367	7.64%	14,801	51.25%	47,168
Married: No	166,477	39.31%	2089	7.23%	168,566
Widowed: No	12,120	2.86%	175	0.61%	12,295
Divorced or Separated: No	38,411	9.07%	837	2.90%	39,248
Never Been Married: No	152,335	35.97%	4437	15.36%	156,772
Urban Residence ×Received SU/MH Treatment					
Large Metropolitan: Yes	30,450	7.19%	5961	20.64%	36,411
Nonmetropolitan: Yes	13,584	3.21%	2947	10.20%	16,531
Small Metropolitan: Yes	27,921	6.59%	6041	20.92%	33,962
Large Metropolitan: No	161,243	38.08%	6034	20.89%	167,277
Nonmetropolitan: No	65,136	15.38%	2661	9.21%	67,797
Small Metropolitan: No	125,130	29.55%	5236	18.13%	130,366
BMI ×Serious Psycological Distress					
Underweight: Yes	1750	0.41%	770	2.67%	2520
Healthy: Yes	18,550	4.38%	7192	24.90%	25,742
Overweight: Yes	13,524	3.19%	4906	16.99%	18,430
Obesity: Yes	13,768	3.25%	5710	19.77%	19,478
Severe Obesity: Yes	4186	0.99%	1893	6.55%	6079
Unknown: Yes	2343	0.55%	871	3.02%	3214
Underweight: No	8194	1.93%	225	0.78%	8419
Healthy: No	120,341	28.42%	2464	8.53%	122,805
Overweight: No	111,008	26.21%	2049	7.09%	113,057
Obesity: No	93,260	22.02%	1934	6.70%	95,194
Severe Obesity: No	19,081	4.51%	524	1.81%	19,605
Unknown: No	17,459	4.12%	342	1.18%	17,801

### FAIR-PLR estimates of suicide ideation/behavior

The FAIR-PLR model produces subgroup-specific regression estimates, allowing each predictor’s effect to vary across demographic and socioeconomic groups. This flexibility captures variations in suicide risk that would otherwise be hidden in a single pooled model.

**Fig. 2:**
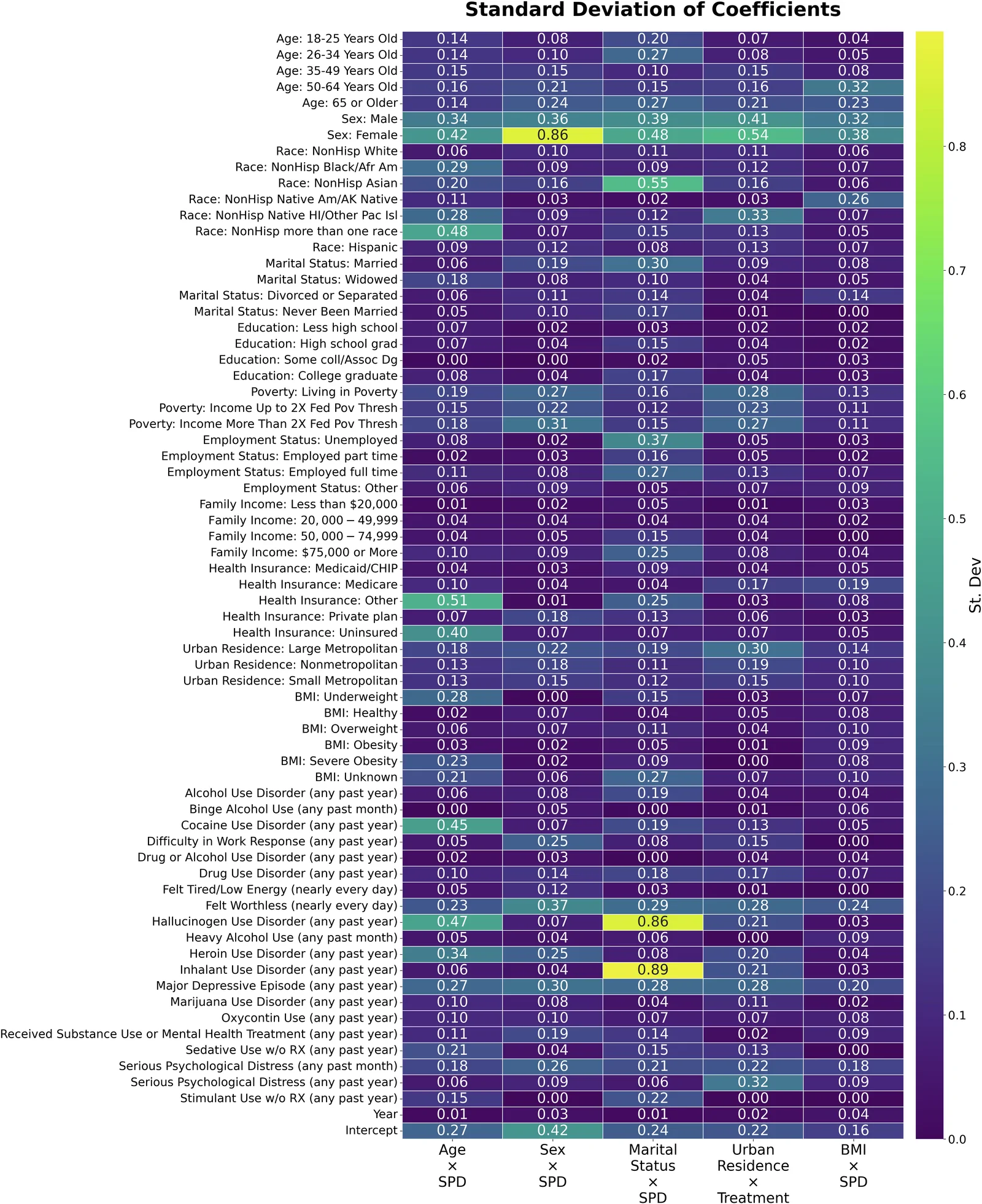
Standard deviation of FAIR-PLR model coefficients across cross-sectional subgroups. Each cell shows the standard deviation of a predictor’s coefficient across the strata of the indicated cross-sectional partition (Age ×SPD, Sex ×SPD, Marital Status ×SPD, Urban Residence ×Treatment, BMI ×SPD); the numerical value is printed inside each cell. Cell color encodes the same value on a viridis scale (color bar at right, range 0.0-0.8), with dark purple indicating low variability, green and teal intermediate variability, and yellow the highest variability. FAIR-PLR functionally adaptive interaction regularization-penalized logistic regression, SPD serious psychological distress, MDE major depressive episode, BMI body mass index, RX prescription.

Figure 2 presents a heatmap of the standard deviation of FAIR-PLR coefficients across cross-sectional subgroups. Each cell shows how strongly a predictor’s effect differs between groups, with warmer colors indicating higher variability. The greatest variation appears for sex, marital status, serious psychological distress (SPD), major depressive episode (MDE), and several substance-use disorders. Substance-use indicators concentrate the largest cross-partition variability in the Marital × SPD axis: inhalant use disorder (*σ* = 0.89) and hallucinogen-use disorder (*σ* = 0.86) shift markedly across marital strata stratified by SPD, while hallucinogen-use disorder is also moderately variable across age strata (*σ* = 0.47 in Age × SPD) and heroin use disorder shows its largest variability in Age × SPD (*σ* = 0.34). Mental-health state indicators reach their largest variability in the Sex × SPD and Urban × Treatment partitions, with “felt worthless” at *σ* = 0.37 in Sex × SPD, MDE at *σ* = 0.31 in Sex × SPD, and SPD (past year) at *σ* = 0.32 in Urban × Treatment. In contrast, the Year variable (*σ* ≤ 0.04), most Family-income levels (*σ* ≤ 0.08), and most BMI categories (*σ* ≤ 0.10) remain low, suggesting stable effects across these subgroups on suicidal outcome.

Supplementary Fig. 2 provides an attribute-level summary of this variability. Demographic and socioeconomic categories show the highest overall variability across single-subgroup axes, with coefficient standard deviations up to 0.76 (Sex — Female) and 0.66 (Poverty — Living in Poverty). Mental-health indicators show moderate variability, with maxima of 0.55 (SPD past year), 0.40 (felt worthless), and 0.38 (MDE), while socioeconomic indicators such as employment and education are stable across these axes (*σ* ≤ 0.18). Substance-use indicators concentrate in specific axes: sedative use without RX shows the largest variability across racial strata (*σ* = 0.71) and heroin use disorder varies most across rurality (*σ* = 0.37). These findings reinforce that mental-health and certain demographic and socioeconomic features contribute most to subgroup divergence in suicide risk.

To interpret variations, subgroup-specific coefficient plots (Supplementary Figs. 14–18) show how effects change for cross-sectional groups such as Age × SPD, Sex × SPD, Marital Status × SPD, Rurality × Treatment, and BMI × SPD. For instance, SPD has strong positive coefficients among females. Female with SPD shows increased risk of suicidal outcome than female without SPD (Supplementary Fig. 15). Younger adults (18–25Y) with hallucinogen-use disorder show slightly elevated risk irrespective of SPD status, whereas adults aged 65 or older with co-occurring SPD show a markedly reduced coefficient (*β* = − 1.55), indicating lower predicted risk in that intersectional stratum (Supplementary Fig. 14). The subgroup-specific coefficient plots (Supplementary Fig. 19) highlight the most influential risk factors within each demographic and socioeconomic category.

Overall, the heatmap in Fig. 2 and Supplementary Fig. 2 highlights where predictor effects vary most strongly across the population, while the supplementary figures reveal which demographic attributes and subgroups drive those differences. These results demonstrate that FAIR-PLR enhances interpretability for subgroup-specific prevention strategies.

### Model performance across subgroups

**Fig. 3:**
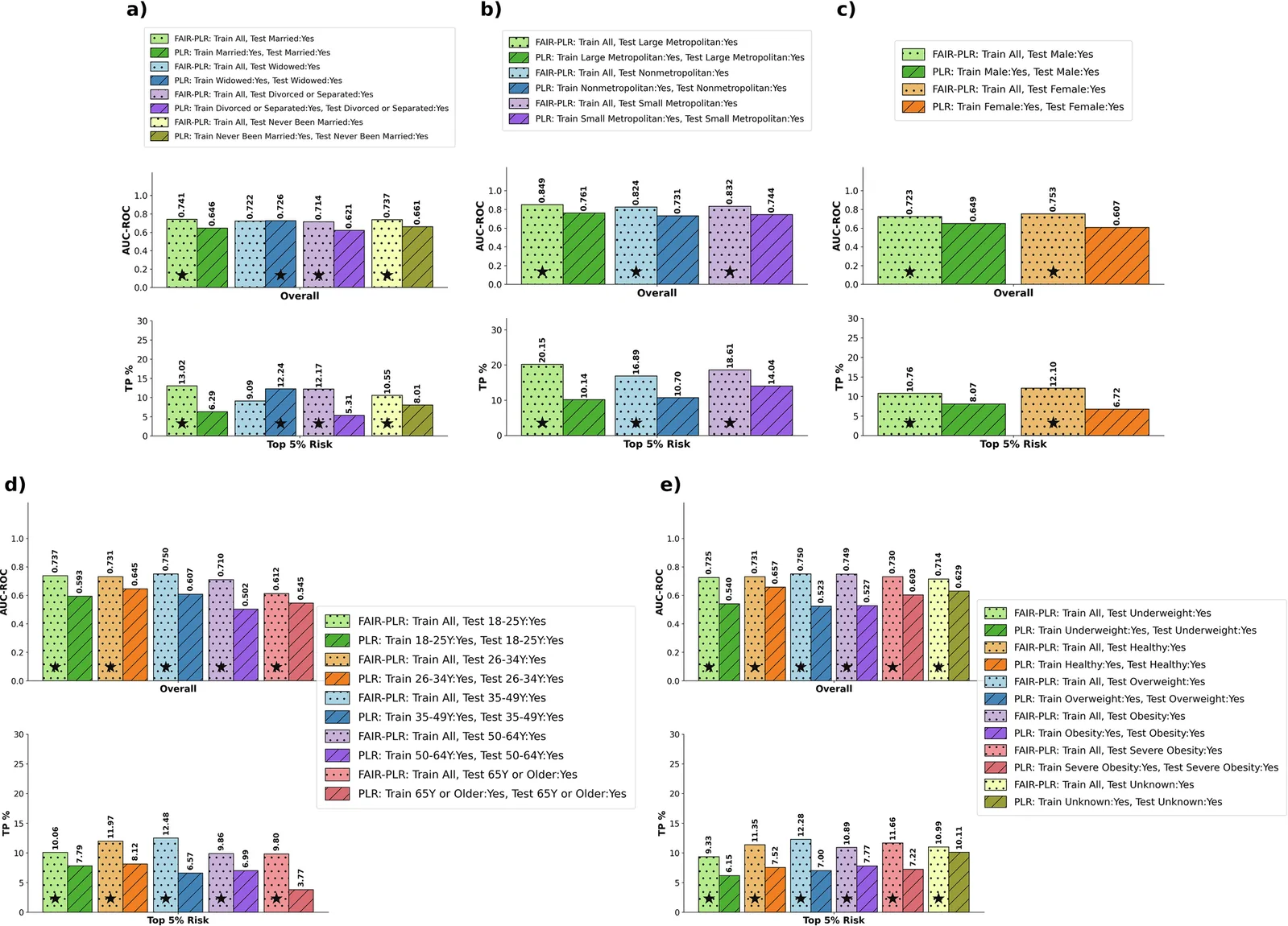
FAIR-PLR matches or exceeds separate per-subgroup PLR within strata with serious psychological distress (SPD) or treatment receipt. Bars show AUC-ROC (left) and true-positive percentage among the top 5% of predicted risk (TP%, right); FAIR-PLR uses dotted fill, per-subgroup PLR uses diagonal-hatch fill, and a black star marks the higher-performing model per stratum. **a** Marital status ×SPD. **b** Rurality ×Treatment. **c** Sex ×SPD. **d** Age ×SPD. **e** BMI ×SPD. All strata restricted to “Yes" (condition present). FAIR-PLR functionally adaptive interaction regularization-penalized logistic regression, PLR penalized logistic regression, SPD serious psychological distress, BMI body mass index, AUC-ROC area under the receiver operating characteristic curve.

**Fig. 4:**
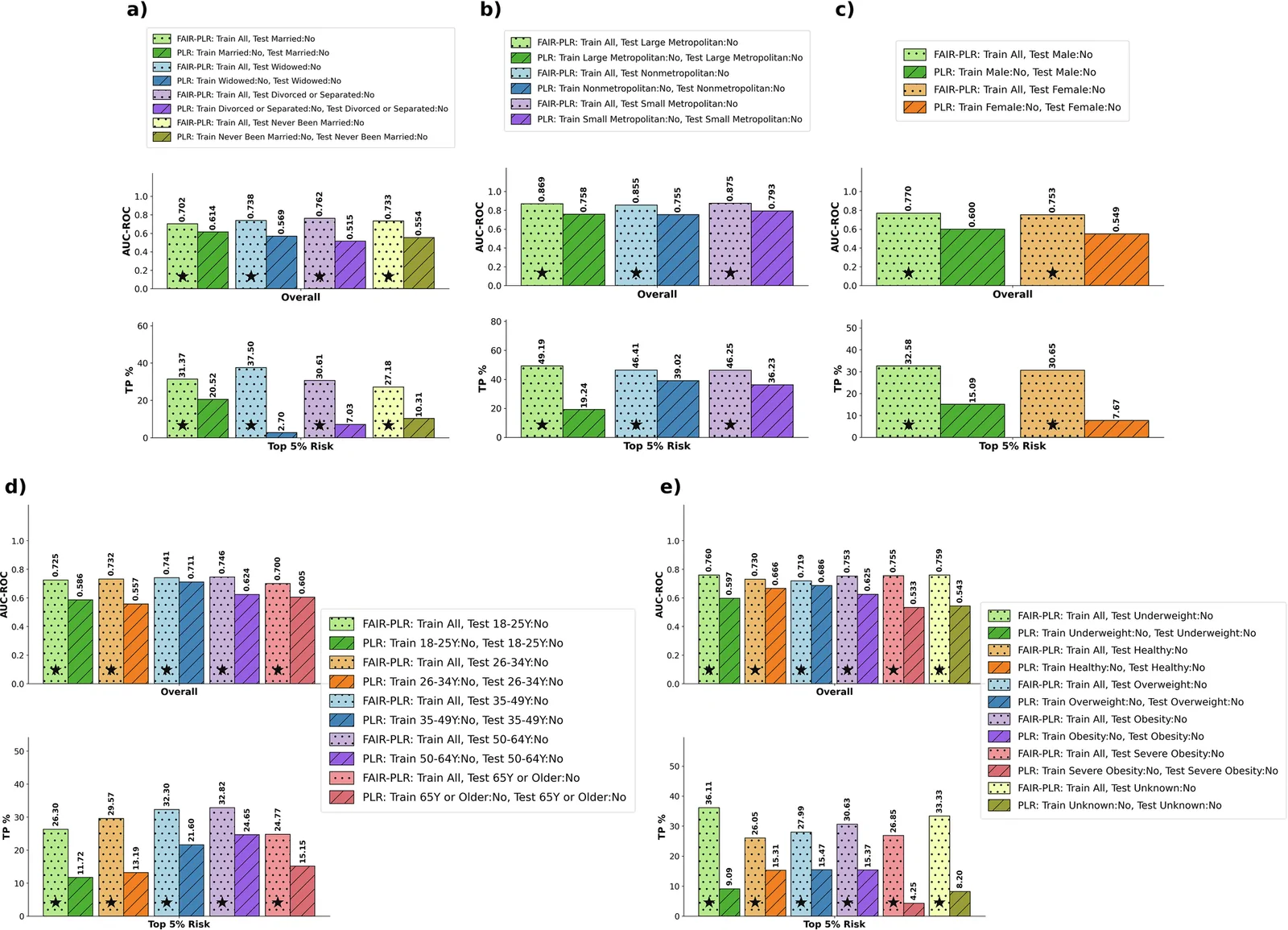
FAIR-PLR gains over separate per-subgroup PLR are largest within strata without serious psychological distress (SPD) or treatment receipt. Bars show AUC-ROC (left) and true-positive percentage among the top 5% of predicted risk (TP%, right); FAIR-PLR uses dotted fill, per-subgroup PLR uses diagonal-hatch fill, and a black star marks the higher-performing model per stratum. **a** Marital status ×SPD. **b** Rurality ×Treatment. **c** Sex ×SPD. **d** Age ×SPD. **e** BMI ×SPD. All strata restricted to “No" (condition absent). FAIR-PLR functionally adaptive interaction regularization-penalized logistic regression, PLR penalized logistic regression, SPD serious psychological distress, BMI body mass index, AUC-ROC area under the receiver operating characteristic curve.

Figures 3, 4 summarize model performance across cross-sectional subgroups defined by serious psychological distress (SPD) with age, BMI, sex, marital status, and mental health treatment with rurality. Overall, FAIR-PLR achieves comparable or superior performance to separate PLR models across nearly all strata, with more pronounced gains among individuals without SPD, suggesting stronger generalization in non-distressed populations. In Fig. 4a, FAIR-PLR shows the largest AUC gains over Separate-PLR for the 26–34Y, 18–25Y, 50–64Y, and 65Y or older groups without SPD (ΔAUC = +0.18, +0.14, +0.12, and +0.10, respectively). With SPD (Figure 3a), the largest gains shift to the 50–64Y, 18–25Y, and 35–49Y groups (ΔAUC = +0.21, +0.14, and +0.14). Similar gains hold for BMI × SPD (where Overweight, Obesity, and Underweight strata with SPD all gain ΔAUC≥ + 0.18) and Marital Status × SPD (where the Divorced or Separated, Never Been Married, and Widowed groups without SPD all gain ΔAUC≥ + 0.17).

In general, FAIR-PLR shows comparable or improved performance over Separate-PLR across the single-subgroup axes (Supplementary Figures 3–13). For Sex ×SPD, FAIR-PLR captures substantially more high-risk cases for both sexes without SPD, with the larger gain on Female:No (+23.0 percentage points of TP at top 5%) than on Male:No (+17.5 points). FAIR-PLR’s improvement is most pronounced on Marital Status ×SPD: Divorced or Separated, Never Been Married, and Widowed groups without SPD gain ΔAUC = + 0.25, + 0.18, and +0.17 respectively, while Married:No gains a smaller +0.09. On the Race axis (Supplementary Fig. 9), the largest AUC gains are for Hispanic (ΔAUC = + 0.33) and Native Hawaiian or Other Pacific Islander (+0.23) respondents, with all seven racial categories improving over Separate-PLR. On the Health Insurance axis (Supplementary Fig. 12), FAIR-PLR’s top-5% TP gain is largest for Private plan (+25.4 points) and Medicaid/CHIP (+21.8 points), with all five insurance categories improving.

Taken together, FAIR-PLR demonstrates robust performance across diverse intersections. The model yields the most significant gains among non-SPD populations while sustaining improved high-risk detection in distressed subgroups. These findings highlight FAIR-PLR’s capability to enhance both overall predictive accuracy and subgroup-specific sensitivity, establishing it as a reliable framework for population-scale suicide risk prediction.

### Differences in model performance across demographic and cross-sectional subgroups

Table 3 report cross-subgroup disparity ratios for the five cross-sectional axes under the combined survey + FAIR weight. These axes pair a demographic variable with a clinical variable (serious psychological distress or treatment receipt) that is itself a predictor in the model, so they characterize effect heterogeneity conditional on clinical state rather than single-subgroup balance. FAIR-PLR attains the smallest Δ_AUC_ on three of five axes, the smallest ${\Delta }_{{\mathrm{TP}}_{5 \% }}$ on two of five, and the smallest ${\Delta }_{{\mathrm{Precision}}_{5 \% }}$ on all five, yielding 10 of 15 cells overall. The cells where FAIR-PLR does not lead have specific structural explanations. Agnostic-PLR attains the smallest Δ_AUC_ on Age × SPD (1.177 vs. 1.225) and on Urban Residence × Treatment (1.060 vs. 1.062); in both cases, the pooled score distribution spreads across axis levels more smoothly when the demographic is not expanded into level-specific interactions, narrowing the AUC ratio even though discrimination within any single level is lower. Separate-PLR attains a smaller ${\Delta }_{{\mathrm{TP}}_{5 \% }}$ on Sex × SPD (2.244 vs. 3.028) because each per-subgroup model is fitted with less effective sample size, which compresses within-subgroup TPR values toward a flat profile; the accompanying ${\Delta }_{{\mathrm{Precision}}_{5 \% }}$ of 16.656 (vs. FAIR-PLR’s 5.762) shows the cost of that flattening. Taken together, the cross-sectional comparison is consistent with the single-subgroup finding reported in Table 4: FAIR-PLR’s discrimination and precision remain the most uniform across intersectional strata, and the competing baselines achieve uniformity on individual metrics only by trading away absolute performance. Table 4 extends this comparison to the six single-subgroup axes (age, sex, race, BMI, health insurance, rurality) for FAIR-PLR, Separate-PLR, and Agnostic-PLR, under the combined survey + FAIR weight. FAIR-PLR achieves the smallest Δ_AUC_ on all 6 axes, the smallest ${\Delta }_{{\mathrm{TP}}_{5 \% }}$ on 4 of 6 axes, and the smallest ${\Delta }_{{\mathrm{Precision}}_{5 \% }}$ on 3 of 6 axes, or 13 of 18 cells overall. Agnostic-PLR takes 5 of the remaining cells and Separate-PLR 0. FAIR-PLR therefore dominates on the cross-subgroup discrimination metric (Δ_AUC_) that the framework is designed to balance while remaining the most consistent performer on threshold-specific capture and precision disparities.

**Table 3:** Cross-sectional subgroup disparity ratios for FAIR-PLR, Separate-PLR, and Agnostic-PLR

Axis	Model	Δ_AUC_	${\Delta }_{{\mathrm{TP}}_{5 \% }}$	${\Delta }_{{\mathrm{Precision}}_{5 \% }}$
Age × SPD	FAIR-PLR	1.225	**3.348**	**10.332**
	Separate-PLR	1.416	6.531	13.985
	Agnostic-PLR	**1.177**	4.838	12.414
Sex × SPD	FAIR-PLR	**1.065**	3.028	**5.762**
	Separate-PLR	1.182	**2.244**	16.656
	Agnostic-PLR	1.093	2.940	6.031
Marital Status × SPD	FAIR-PLR	**1.085**	**4.125**	**8.492**
	Separate-PLR	1.410	7.591	61.509
	Agnostic-PLR	1.167	4.370	10.824
Urban Residence × Treatment	FAIR-PLR	1.062	2.912	**1.901**
	Separate-PLR	1.085	3.848	3.696
	Agnostic-PLR	**1.060**	**2.536**	2.612
BMI × SPD	FAIR-PLR	**1.064**	3.869	**6.716**
	Separate-PLR	1.312	3.636	29.724
	Agnostic-PLR	1.237	**3.496**	13.922

Each cell is the max/min ratio of the metric across subgroup levels within the axis; bold marks the smallest disparity per axis ×metric. Cross-sectional axes pair a demographic variable with a clinical variable (SPD or treatment receipt) that is also a predictor in the model, so these axes characterize effect heterogeneity conditional on clinical state rather than single-subgroup balance.

**Table 4:** Cross-subgroup performance disparity across six single-subgroup axes for FAIR-PLR, Separate-PLR, and Agnostic-PLR

Axis	Model	Δ_AUC_	${\Delta }_{{\mathrm{TP}}_{5 \% }}$	${\Delta }_{{\mathrm{Precision}}_{5 \% }}$
Age	FAIR-PLR	**1.144**	1.769	4.027
	Separate-PLR	1.395	2.798	4.947
	Agnostic-PLR	1.270	**1.728**	**3.678**
Sex	FAIR-PLR	**1.009**	1.095	**1.092**
	Separate-PLR	1.028	2.220	2.615
	Agnostic-PLR	1.029	**1.062**	1.252
Race	FAIR-PLR	**1.055**	**1.598**	1.574
	Separate-PLR	1.416	2.304	2.779
	Agnostic-PLR	1.121	2.704	**1.474**
BMI	FAIR-PLR	**1.027**	**1.485**	**1.260**
	Separate-PLR	1.193	1.636	2.810
	Agnostic-PLR	1.029	2.429	1.918
Health Insurance	FAIR-PLR	**1.019**	**1.409**	1.807
	Separate-PLR	1.129	3.439	1.951
	Agnostic-PLR	1.110	1.832	**1.337**
Rurality	FAIR-PLR	**1.016**	**1.085**	**1.059**
	Separate-PLR	1.144	1.338	1.184
	Agnostic-PLR	1.025	1.158	1.111

Each cell is the max/min ratio of the metric across subgroup levels within the axis; lower values indicate smaller subgroup gaps. Bold marks the smallest disparity per axis ×metric. All three models were retrained under the combined survey + FAIR weight of Eq. (4).

**Table 5:** Overall performance of FAIR-PLR and three baselines (Standard logistic regression; Pooled elastic-net with group–covariate interactions and uniform observation weights; Decision Tree) on the held-out test set, broken down by single-subgroup axis

Subgroup axis	Model	AUC-ROC [95% CI]	TPR@5% [95% CI]	Precision@5% [95% CI]	DeLong *p*
Age	FAIR-PLR	0.885 [0.880, 0.890]	0.388 [0.378, 0.398]	0.495 [0.483, 0.508]	—
	Standard LR	0.885 [0.881, 0.890]	0.387 [0.378, 0.398]	0.495 [0.483, 0.508]	0.564
	Pooled EN (no weight)	0.886 [0.881, 0.890]	0.388 [0.378, 0.398]	0.495 [0.483, 0.508]	< 0.001
	Decision Tree	0.882 [0.877, 0.886]	0.387 [0.380, 0.402]	0.494 [0.485, 0.513]	< 0.001
Gender	FAIR-PLR	0.886 [0.881, 0.890]	0.388 [0.378, 0.398]	0.495 [0.483, 0.508]	—
	Standard LR	0.885 [0.881, 0.890]	0.387 [0.378, 0.398]	0.495 [0.483, 0.508]	0.130
	Pooled EN (no weight)	0.887 [0.882, 0.891]	0.389 [0.379, 0.399]	0.497 [0.484, 0.510]	0.001
	Decision Tree	0.882 [0.877, 0.886]	0.387 [0.380, 0.402]	0.494 [0.485, 0.513]	< 0.001
Race	FAIR-PLR	0.881 [0.876, 0.885]	0.391 [0.381, 0.401]	0.500 [0.487, 0.512]	—
	Standard LR	0.885 [0.881, 0.890]	0.387 [0.378, 0.398]	0.495 [0.483, 0.508]	< 10^−6^
	Pooled EN (no weight)	0.887 [0.882, 0.891]	0.390 [0.380, 0.400]	0.498 [0.486, 0.511]	< 10^−6^
	Decision Tree	0.882 [0.877, 0.886]	0.387 [0.380, 0.402]	0.494 [0.485, 0.513]	0.264
BMI	FAIR-PLR	0.885 [0.880, 0.889]	0.387 [0.378, 0.399]	0.494 [0.483, 0.510]	—
	Standard LR	0.885 [0.880, 0.889]	0.387 [0.380, 0.400]	0.495 [0.485, 0.511]	0.045
	Pooled EN (no weight)	0.886 [0.881, 0.890]	0.389 [0.380, 0.399]	0.497 [0.485, 0.510]	< 0.001
	Decision Tree	0.882 [0.878, 0.886]	0.387 [0.378, 0.400]	0.494 [0.483, 0.511]	0.004
Health Insurance	FAIR-PLR	0.885 [0.880, 0.889]	0.389 [0.379, 0.398]	0.497 [0.485, 0.508]	—
	Standard LR	0.885 [0.880, 0.890]	0.387 [0.378, 0.397]	0.495 [0.483, 0.507]	0.045
	Pooled EN (no weight)	0.886 [0.881, 0.891]	0.388 [0.379, 0.398]	0.496 [0.484, 0.508]	< 0.001
	Decision Tree	0.882 [0.876, 0.886]	0.387 [0.378, 0.401]	0.494 [0.482, 0.512]	0.002
Urban Residence	FAIR-PLR	0.885 [0.880, 0.890]	0.386 [0.377, 0.396]	0.493 [0.481, 0.506]	—
	Standard LR	0.885 [0.880, 0.890]	0.387 [0.378, 0.397]	0.495 [0.483, 0.507]	0.862
	Pooled EN (no weight)	0.886 [0.881, 0.891]	0.387 [0.378, 0.397]	0.494 [0.482, 0.507]	0.014
	Decision Tree	0.882 [0.876, 0.886]	0.387 [0.378, 0.401]	0.494 [0.482, 0.512]	< 0.001

FAIR-PLR is trained under the combined survey + FAIR weight of equation (4); Standard-LR and Decision-Tree use the NSDUH analysis weight rescaled as ANALWT/11; Pooled-EN-no-weight is deliberately unweighted to isolate the contribution of the subgroup-size weighting. Each cell reports the point estimate with a 95% stratified bootstrap CI (1000 iterations). The DeLong column gives the two-sided *p*-value for the AUC difference vs. FAIR-PLR.

Table 5 reports overall test-set AUC, TPR@5%, and Precision@5% with 95% stratified bootstrap confidence intervals and DeLong two-sided *p*-values for FAIR-PLR against each baseline. FAIR-PLR’s AUC falls within 0.005 of the strongest pooled baseline (Pooled-EN-no-weight) on five of six axes. On the Race axis, FAIR-PLR’s AUC decreases to 0.881 [0.876, 0.885] against 0.887 [0.882, 0.891] for Pooled-EN-no-weight; this 0.006 decrease accompanies the Race-axis subgroup-balance gain reported in Table 4, where FAIR-PLR’s Δ_AUC_ falls to 1.055 from 1.416 under Separate-PLR. FAIR-PLR therefore matches or nearly matches global discrimination while substantially reducing cross-subgroup AUC disparity.

## Discussion

This study presents FAIR-PLR as an interpretable, subgroup-optimized framework for suicide risk stratification using eleven years of NSDUH data. FAIR-PLR consistently matches or exceeds the performance of separate penalized logistic regression models across demographic and socioeconomic subgroups, including sex, age, race and ethnicity, BMI, rurality, and insurance status. It also narrows subgroup performance gaps, particularly for small or underrepresented groups such as Native Hawaiian or Other Pacific Islander respondents, while retaining transparency through subgroup-specific coefficients. These findings position FAIR-PLR as a robust and explainable tool for national-scale suicide risk prediction.

Beyond single-attribute analysis, cross-sectional experiments show that FAIR-PLR maintains balanced performance when demographic, socioeconomic, and psychological factors intersect. Across combinations such as age × SPD, marital status × SPD, BMI × SPD, and rurality ×treatment, the cross-subgroup precision gap at the top 5% threshold (${\Delta }_{{\mathrm{Precision}}_{5 \% }}$) decreased by 49–86% relative to Separate-PLR (largest reductions on Marital × SPD, −86%, and BMI ×SPD, −77%), and the AUC gap (Δ_AUC_) decreased on four of five cross-sectional axes (Table 3). Consistent with prior work, reducing bias improves performance^17^, especially for underrepresented groups with high true-positive error rates. FAIR-PLR also identifies suicidal cases without distress that separate models miss, revealing patterns traditional approaches overlook.

Explicit modeling of subgroup-covariate interactions captures heterogeneity in how risk factors operate across populations. SPD shows stronger effects for females, younger adults, and those with healthy BMI (Table 2), aligning with prior NSDUH evidence of elevated suicidal vulnerability in these groups^21,22^. Coley et al.^11^ reported standard predictive models performed well overall but poorly for Black and American Indian/Alaska Native patients, mirroring our poor outcome from a separate PLR model on non-Hispanic native HI/other pacific islander subgroup (Supplementary Fig. 9). By assigning subgroup-specific interactions and weight adjustments, FAIR-PLR mitigates such differences in performance, improving detection among small or historically marginalized groups. Quantitatively, the cross-subgroup TPR ratio at the top 5% threshold decreased from 6.531 to 3.348 within Age ×SPD strata and from 7.591 to 4.125 within Marital Status ×SPD when moving from Separate-PLR to FAIR-PLR, demonstrating substantial reductions in intersectional disparities (Table 3).

The cross-sectional findings further clarify that suicide risk arises from interacting rather than isolated determinants. SPD effects intensified suicidal behavior among unmarried adults compared to other marital status category (Table 2). This suggests that mental distress compounds social risk factors such as loneliness and reduced emotional support, thereby elevating suicide vulnerability across relational contexts^32^. Suicide risk remained elevated among individuals in small metropolitan and non-metropolitan areas (Table 2), and limited treatment access in rural regions reflected persistent structural inequities in mental-health care availability^33^. Several of these interaction effects line up with mechanism-level accounts that go beyond statistical association. The stronger SPD effect in 18–25-year-olds likely reflects developmental vulnerability at a life stage with incomplete coping strategies and historically lower engagement with formal mental-health services. The higher SPD effect in females tracks the documented sex asymmetry between suicidal ideation prevalence and attempt lethality, and flags a risk: screening tools calibrated on higher-lethality male profiles may underweight ideation-dominant presentations in women. The rurality ×limited-treatment pattern lines up with structural drivers such as mental-health-provider shortages in non-metropolitan counties, longer travel times to care, and community-level stigma in smaller populations. These readings are hypotheses generated by FAIR-PLR’s subgroup-specific coefficients, not causal claims, given the cross-sectional design of NSDUH.

Subgroup-aware frameworks like FAIR-PLR improve interpretability, addressing the growing demand for explainable AI in suicide-risk modeling^12^. Unlike opaque machine-learning models, FAIR-PLR offers transparent coefficients that illustrate how factors such as depression, substance use, and social context interact to influence suicide risk across different subgroups. The model’s subgroup-level performance gains reflect national trends in rising suicidality among specific populations^34^, underscoring its utility in identifying high-risk groups with greater precision.

FAIR-PLR’s outputs map onto existing public-health and clinical workflows through two threshold tiers: the top 1% of predicted risk flags candidates for intensive clinical review or same-week outreach, and the top 5% defines a broader screening tier for proactive telephone follow-up or community-level programs. Public-health agencies such as SAMHSA and the Department of Veterans Affairs can use the subgroup-specific coefficients to see which risk factors matter most within each demographic stratum, and to direct prevention resources (clinician hiring, helpline capacity, outreach campaigns) toward subgroups with the largest unaddressed risk. FAIR-PLR’s linear form also admits coefficient-level updates for EHR-integrated decision support as new data accrue. To avoid drift, any operational deployment should include quarterly recalibration and a standing subgroup-level audit that re-checks TPR and Precision at the top-risk thresholds on the most recent cohort; coefficient changes beyond a pre-specified tolerance should trigger review rather than a silent model update. On the governance side, the subgroup-equal loss property makes FAIR-PLR a natural fit for settings where disparate-impact audits are required by policy, since the training objective constrains cross-subgroup aggregate loss to be equal rather than leaving the balance to post-hoc calibration.

This study’s primary strengths include the integration of a decade of harmonized NSDUH data, yielding one of the largest interpretable suicide-risk modeling analyses to date, with over 450,000 adult participants. The cross-sectional interaction analysis offers an intersectional approach to suicide risk that has been rarely explored in prior studies. Additionally, rigorous evaluation metrics, such as AUC-ROC and true-positive percentages in top-risk strata, provide a robust assessment of the model’s ability to identify high-risk individuals. Nevertheless, several limitations remain. First, reliance on self-reported survey data introduces potential recall and reporting biases, particularly for sensitive variables^35^. Second, the use of penalized logistic regression may inflate coefficient magnitudes due to regularization effects when predictors are highly correlated. Third, the NSDUH dataset lacks detailed clinical and genetic information that could enhance prediction accuracy, and the cross-sectional design limits our ability to make causal inferences or analyze temporal risk trajectories. Fourth, external validity is bounded by the NSDUH sampling frame: the household-based design excludes unsheltered homeless individuals, incarcerated populations, and active-duty military personnel, each of which carries elevated suicide risk. Coefficients learned from the NSDUH cohort should not be transferred to these populations without revalidation. Fifth, the 2013–2023 window spans two NSDUH methodological redesigns: the 2020 collection was disrupted by COVID-19 and used a Quarters 1 and 4 weight (ANALWTQ1Q4_C), and the 2021–2023 collections added a web-interview mode with a revised weight (ANALWT2_C). We pool the three era-specific weights into one column and apply SAMHSA’s canonical multi-year rescaling (division by *Y* = 11) so that the pooled weights sum to a single-year-equivalent U.S. population; this lets the combined weight (4) be used on the full eleven-year sample. Some residual composition drift between the pre- and post-redesign frames is not fully removed by rescaling alone. Sixth, suicide-risk prediction carries ethical risks that better subgroup performance alone does not resolve: false-positive flags impose a real burden on individuals and clinicians; visible subgroup-level risk estimates can be misused to restrict rather than expand access to care ("risk-based gatekeeping”); and public reporting of subgroup prevalences can reinforce stigma toward the highest-risk groups. Any deployment of FAIR-PLR should therefore come with an equity impact assessment, clinician-in-the-loop workflows that keep risk scores advisory rather than deterministic, and ongoing public reporting of subgroup-level true-positive and false-positive rates.

Methodologically, FAIR-PLR shows how adaptations of classical statistical models can link machine learning advances with subgroup risk modeling. Its transparency and low computational burden make it suitable for survey, clinical, and policy contexts where interpretability is essential. As a hybrid approach that combines statistical rigor with flexible interactions, FAIR-PLR improves subgroup prediction without losing explainability. This balance of precision and clarity makes it a strong option for public health research, especially when stakeholder trust is important.

FAIR-PLR can be applied to diverse datasets, and its FAIR approach can be adapted to many ML models. By identifying subgroup-specific patterns, it provides actionable insights for improving individual care and population health. Public health agencies can use it to detect emerging high-risk groups, monitor shifts in suicide vulnerability, and tailor interventions. The model can also support clinical decision systems by helping providers identify individuals who may benefit from targeted mental health care. FAIR-PLR has strong policy implications: it can guide more effective allocation of prevention resources by quantifying suicide risk at the subgroup level. Its transparent, subgroup-specific coefficients allow policymakers to justify decisions with evidence-driven insights. Practitioners can use these interpretable patterns to understand key drivers of suicide risk and better target community interventions.

The main FAIR-PLR fit already folds NSDUH’s person-level survey weight into the training loss via equation (4), so the reported coefficients and subgroup performance reflect a nationally representative rather than sample-representative objective.

Future work can extend this study in several ways. External validation using clinical datasets, including electronic health records or national registries, would improve generalizability and test FAIR PLR in real-world settings, while helping address limitations of self-reported NSDUH data. Longitudinal methods such as survival analysis should be explored to capture temporal changes in suicide risk. More granular intersectional analyses that consider social determinants, community factors, and access to care may reveal additional drivers of vulnerability and support population-specific prevention strategies. Finally, incorporating features such as genetic markers, biomarkers, or treatment response data could enhance predictive accuracy and deepen understanding of the biological factors linked to suicide risk.

## Supplementary Information

Supplementary Information

## Data Availability

Data used in this study are publicly available from the Substance Abuse and Mental Health Services Administration (SAMHSA) through the National Survey on Drug Use and Health (NSDUH) data portal https://www.samhsa.gov/data/data-we-collect/nsduh-national-survey-drug-use-and-health/datafiles.

## Code availability

The code used for data harmonization, modeling, and analysis is available at https://github.com/sharminafrose/FAIR-PLR_Suicide-Risk.
