# Supplementary material for: Subgroup-aware suicide risk prediction via functionally adaptive interaction regularized regression
Source: Npj Ment Health Res. 2026 Jul 22;5:56. doi: 10.1038/s44184-026-00229-y (PMC13760047; doi:10.1038/s44184-026-00229-y)
Supplement: Supplementary file 1 [file 44184_2026_229_MOESM1_ESM.pdf]

Subgroup-Aware Suicide Risk Prediction via Functionally Adaptive Interaction Regularized Regression

Supplementary Materials

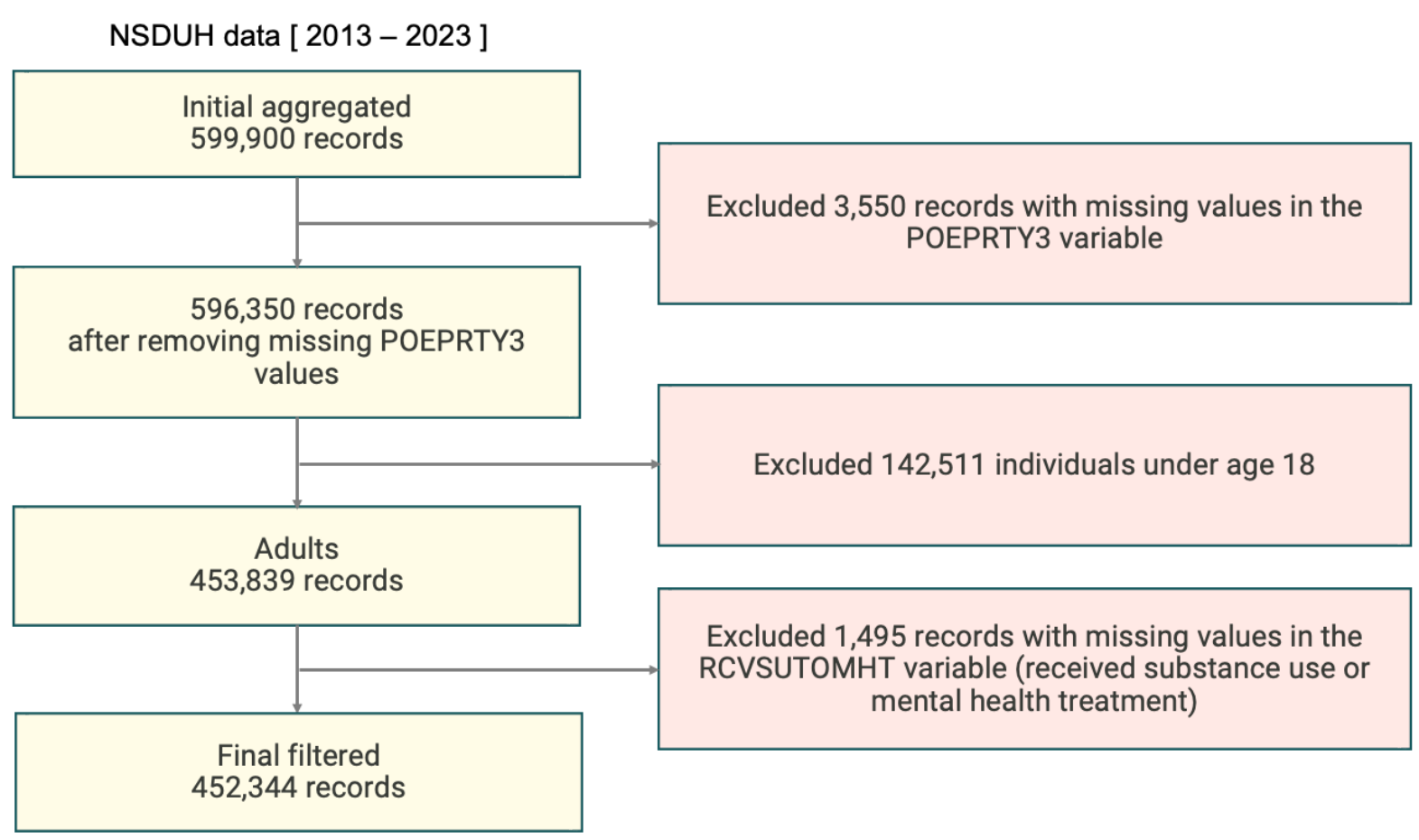

Supplementary Figure 1: **Cohort construction flow diagram from raw NSDUH input to the final analytical sample.** Yellow rectangular boxes (read top to bottom) show successive sample sizes, and pink rectangular boxes on the right list the exclusion criterion applied at each step: removal of 3,550 records with missing POEPRTY3, exclusion of 142,511 individuals under age 18, and removal of 1,495 records with missing RCVSUTOMHT (received substance use or mental health treatment), yielding a final analytical sample of 452,344 records. Abbreviations: NSDUH = National Survey on Drug Use and Health.

Supplementary Table 1: Variable coding harmonization across NSDUH survey years (2013–2023), showing adjustments to variable names and values to ensure consistency in key predictors and in the NSDUH person-level analysis weight used by FAIR-PLR's combined survey+FAIR weight

**Predictors**	**2023-2022**	**2021**	**2020**	**2019 - 2016**	**2015**	**2014-2013**
RCVSUTOMHT	1 if RCVSUTOMHT = 1 otherwise 0	1 if RCVMHOSPTX4 = 1 otherwise 0	1 if (AMHTXRC3 = 1 OR TXYRALDGB1 in {1,2}) otherwise 0			1 if (AMHTXRC3 = 1 OR TXYRADG in {1,2}) otherwise 0
UD5ILLANY	No change		UDYR5ILL	ILLYR	ILLYR	SUMYR
UD5ILALANY	No change		UDYR5ILAL	ILLORALC		ILORALC
SPDPSTMON	No change		SPDMON			
SPDPSTYR	No change		SPDYR			
IRIMPRESP	1 IRIMPRESP in {2,3,4,5} otherwise 0		1 IMPRESP in {2,3,4,5} otherwise 0			
ADSUITPAYR	1 if ADSUITPAYR = 1 otherwise 0		1 if (MHSUITHK = 1 OR MHSUIPLN =1 OR MHSUITRY = 1) otherwise 0			
SURVEY_WEIGHT	ANALWT2_C		ANALWTQ1Q4_C	ANALWT_C		
IRPYUD5ALC	No change			ALCYR		
IRPYUD5MRJ	No change			MRJYR		
IRPYUD5COC	No change			COCYR	COCYR	COCYR
IRPYUD5HER	No change			HERYR	HERYR	HERYR
IRPYUD5HAL	No change			HALLUCYR	HALLUCYR	HALYR
IRPYUD5INH	No change			INHALYR	INHALYR	INHYR
IRMARIT	No change				IRMARITSTAT	No change
STMWYNORX	1 if STMWYNORX == 1 otherwise 0				1 if STDAYPYR in [1, 365] otherwise 0	
SEDWYNORX	1 if SEDWYNORX == 1 otherwise 0					1 SEDYRTOT in [1, 365] otherwise 0
COUTYP4	No change					COUTYP2
EDUHIGHCAT	No change					EDUCCAT2
IRWRKSTAT18	No change					EMPSTAT4
OXYCNANYYR	No change					OXYYR
BNGDRKMON	No change					BINGEDRK
HVYDRKMON	No change					HVYDRK2
POVERTY3	No change					POVERTY2
AD_MDEA6	1 if AD_MDEA6 = 1 otherwise 0					
AD_MDEA7	1 if AD_MDEA7 = 1 otherwise 0					
IRINSUR4	1 IRINSUR4 = 1 otherwise 0					
CATAG6	No change					
IRSEX	No change					
BMI2	No change					
NEWRACE2	No change					
AMDEYR	1 if AMDEYR = 1 otherwise 0					
INCOME	No change					
Health_Coverage	1 if IRPRVHLT = 1 else 2 if IRMCDCHP = 1 else 3 if IRMEDICR = 1 else 4 if IROTHHLT = 2 else 5					

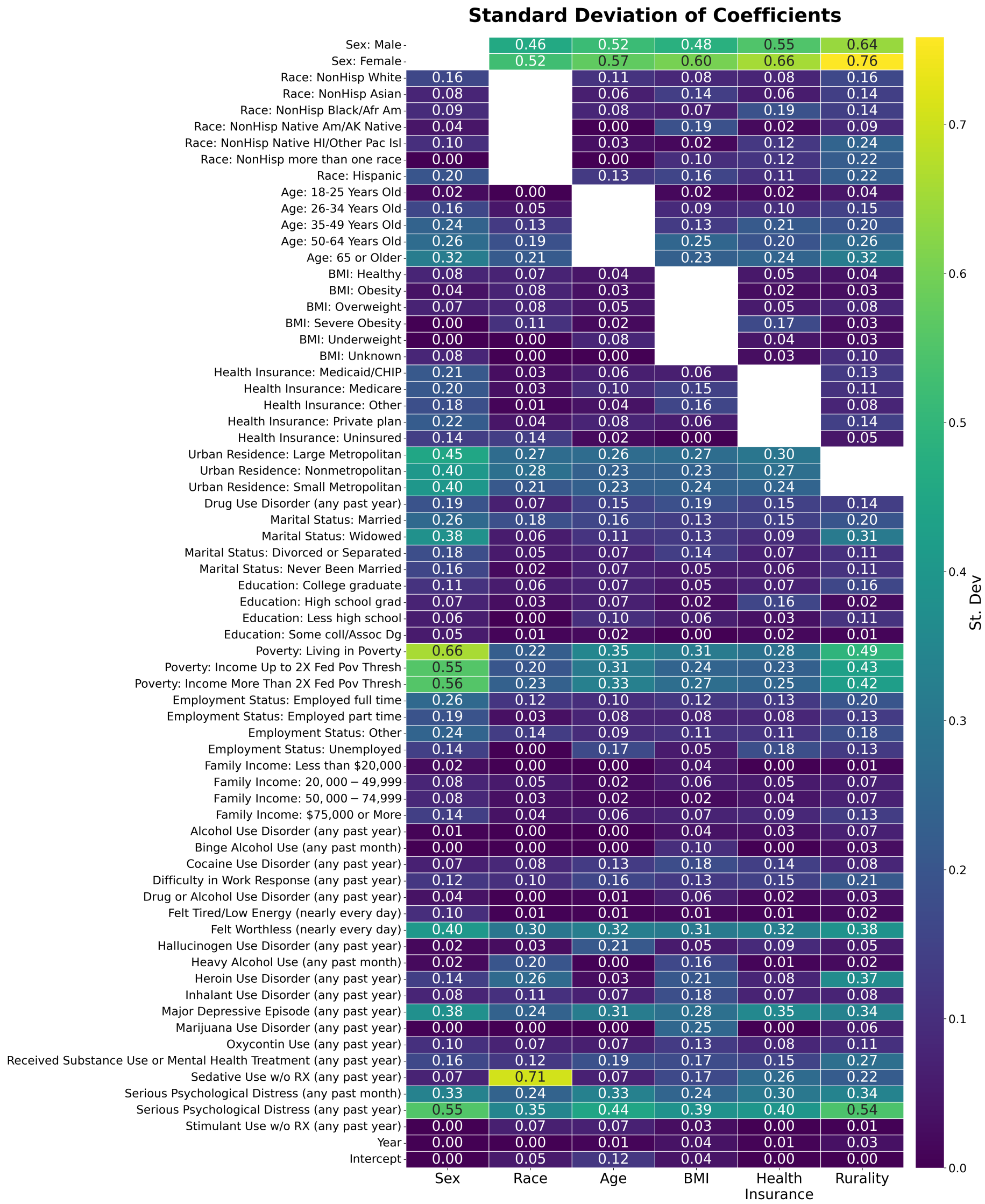

Supplementary Figure 2: **Standard deviation of FAIR-PLR model coefficients across single-subgroup axes (Sex, Race, Age, BMI, Health Insurance, Rurality).** Each cell shows the standard deviation of a predictor's coefficient across the levels of the indicated subgroup axis, with the numerical value printed inside the cell; cell color encodes the same value on a viridis scale (color bar at right), with dark purple indicating low variability, green and teal intermediate variability, and yellow the highest variability. Abbreviations: FAIR-PLR = functionally adaptive interaction regularization–penalized logistic regression; BMI = body mass index.

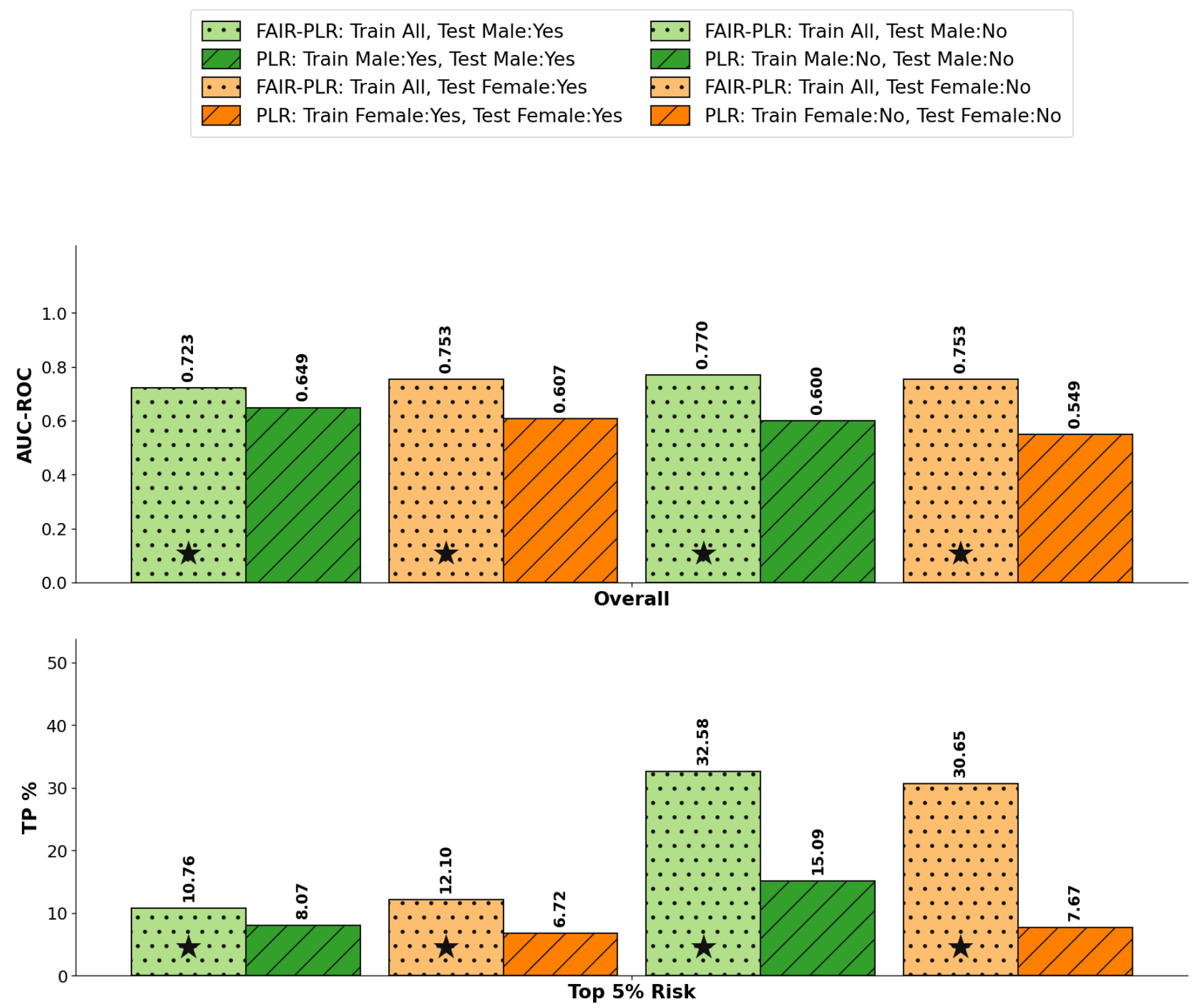

Supplementary Figure 3: **Performance of FAIR-PLR and separate PLR models across cross-sectional subgroups defined by sex and serious psychological distress (SPD) status.** The top panel shows AUC-ROC and the bottom panel shows true-positive percentage (TP%) at the top 5% of predicted risk. Each FAIR-PLR bar (dotted fill) represents the single FAIR-PLR model trained on the full sample and evaluated on one test stratum (for example, Male:Yes denotes males with SPD); each PLR bar (diagonal-hatch fill) represents a separate PLR fit trained and evaluated within that same stratum. Colors distinguish strata as shown in the legend, and a black star marks the higher-performing model within each stratum. Females with SPD form the largest subgroup (43.5%). Abbreviations: FAIR-PLR = functionally adaptive interaction regularization–penalized logistic regression; PLR = penalized logistic regression; SPD = serious psychological distress; AUC-ROC = area under the receiver operating characteristic curve; TP% = true-positive percentage.

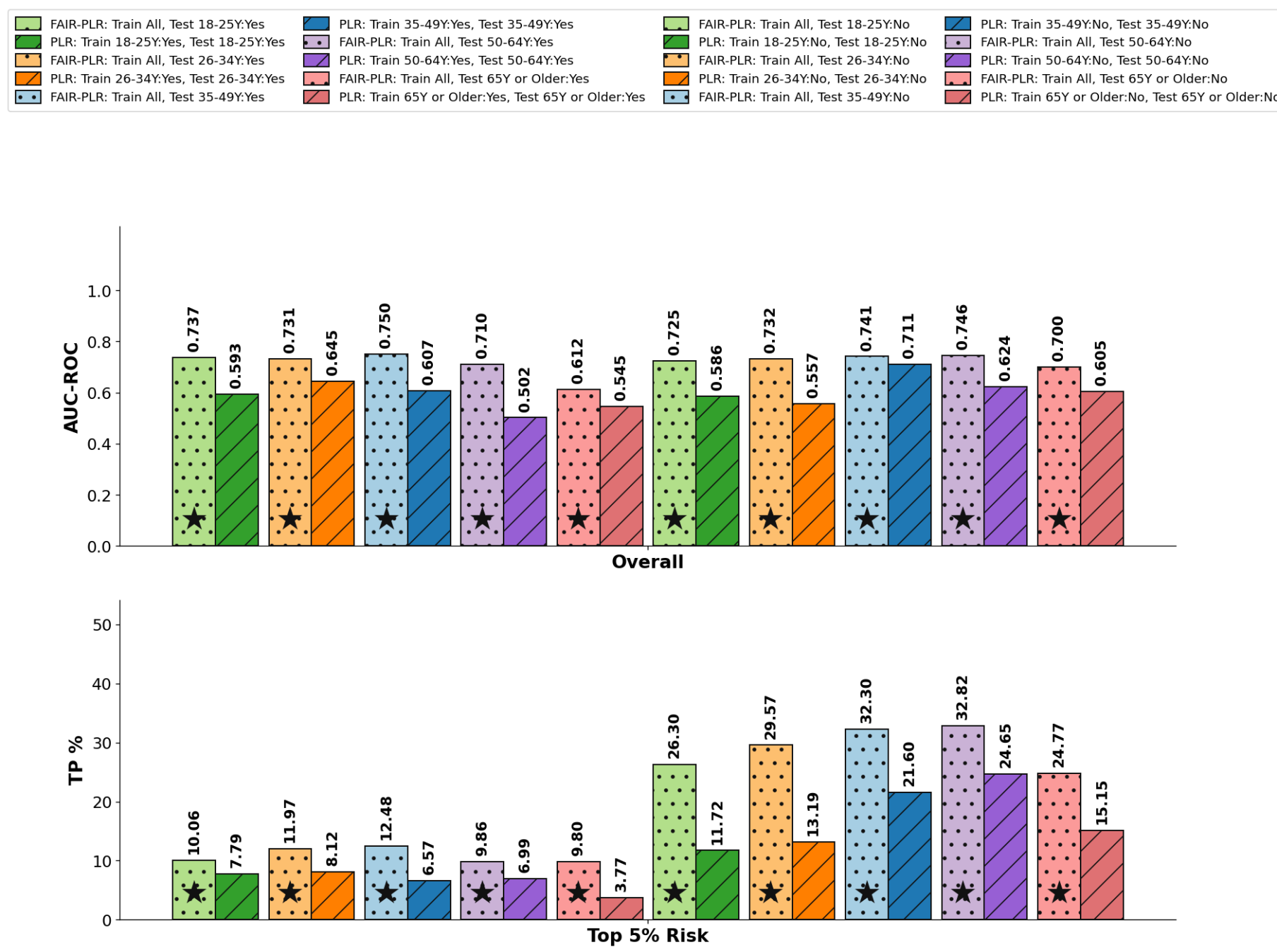

Supplementary Figure 4: **Performance of FAIR-PLR and separate PLR models across cross-sectional subgroups defined by age and serious psychological distress (SPD) status.** The top panel shows AUC-ROC and the bottom panel shows true-positive percentage (TP%) at the top 5% of predicted risk. Bars compare FAIR-PLR (dotted fill) and per-subgroup PLR (diagonal-hatch fill); colors distinguish age $\times$ SPD strata as shown in the legend, and a black star marks the higher-performing model within each stratum. Adults aged 18–25 with SPD form the largest subgroup (24.1%). Abbreviations: FAIR-PLR = functionally adaptive interaction regularization–penalized logistic regression; PLR = penalized logistic regression; SPD = serious psychological distress; AUC-ROC = area under the receiver operating characteristic curve; TP% = true-positive percentage.

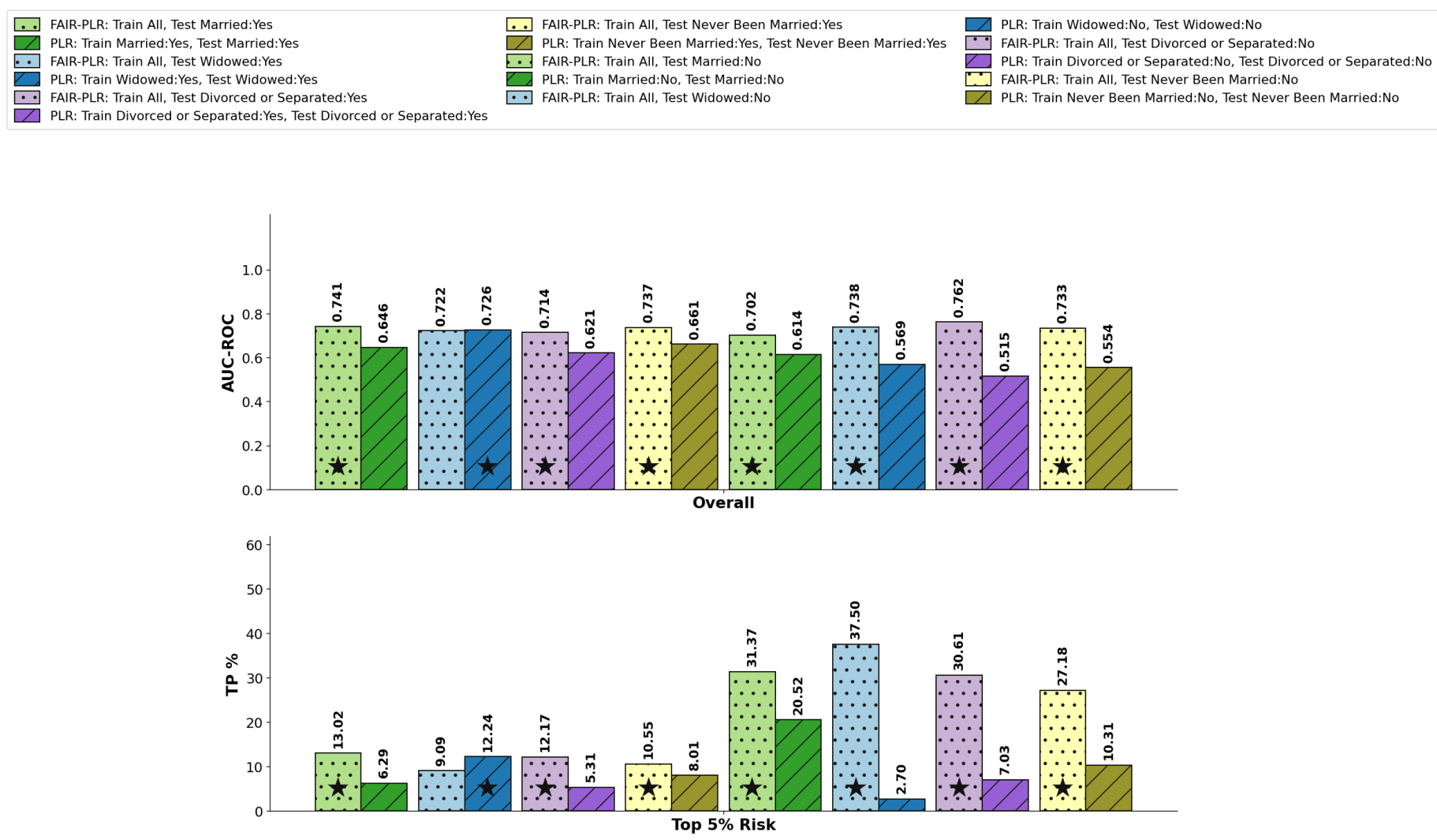

Supplementary Figure 5: **Performance of FAIR-PLR and separate PLR models across cross-sectional subgroups defined by marital status and serious psychological distress (SPD) status.** The top panel shows AUC-ROC and the bottom panel shows true-positive percentage (TP%) at the top 5% of predicted risk. Bars compare FAIR-PLR (dotted fill) and per-subgroup PLR (diagonal-hatch fill); colors distinguish marital status $\times$ SPD strata as shown in the legend, and a black star marks the higher-performing model within each stratum. Never-married individuals with SPD form the largest subgroup (37.3%). Abbreviations: FAIR-PLR = functionally adaptive interaction regularization–penalized logistic regression; PLR = penalized logistic regression; SPD = serious psychological distress; AUC-ROC = area under the receiver operating characteristic curve; TP% = true-positive percentage.

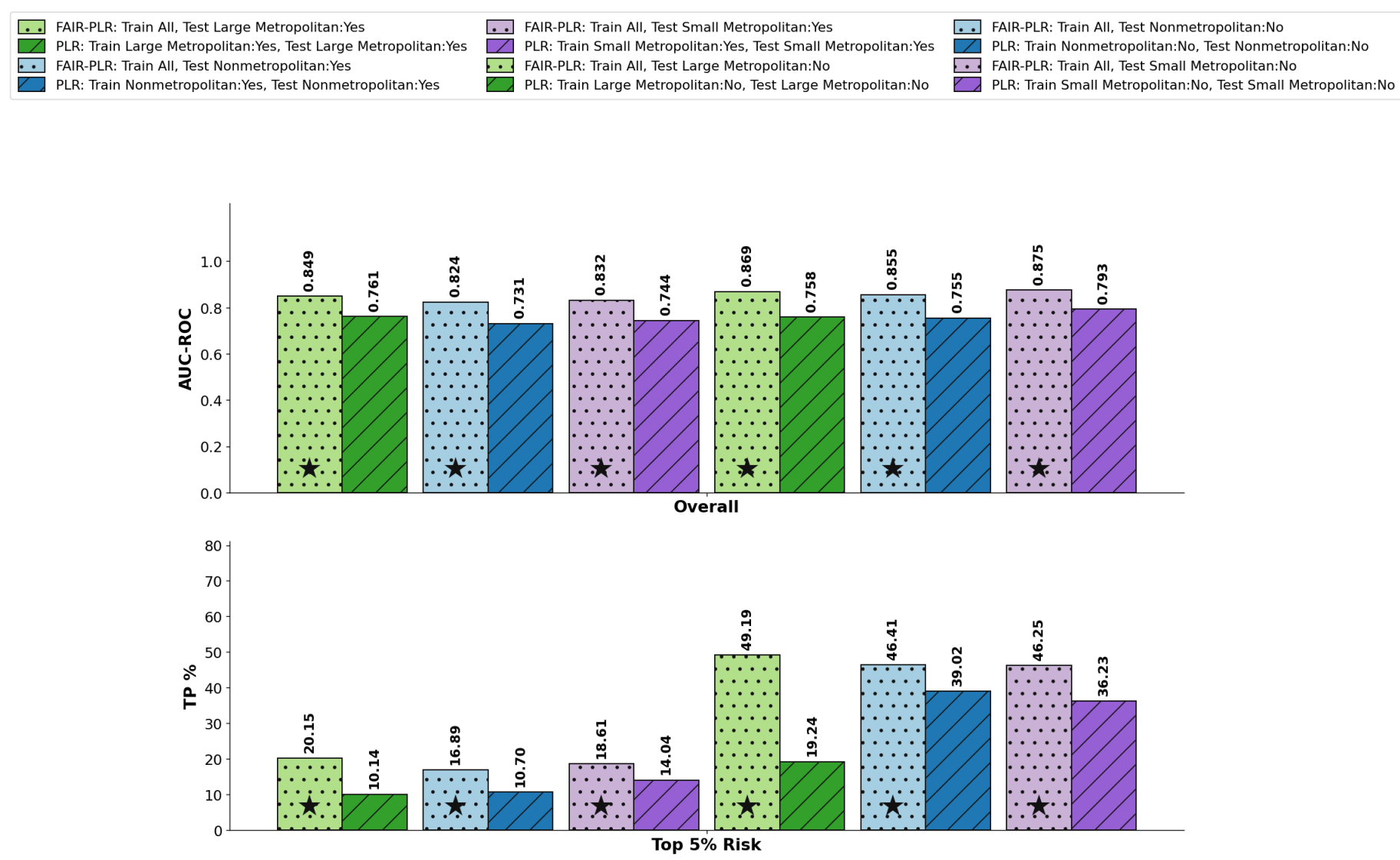

Supplementary Figure 6: **Performance of FAIR-PLR and separate PLR models across cross-sectional subgroups defined by rurality and receipt of substance-use or mental-health treatment.** The top panel shows AUC-ROC and the bottom panel shows true-positive percentage (TP%) at the top 5% of predicted risk. Bars compare FAIR-PLR (dotted fill) and per-subgroup PLR (diagonal-hatch fill); colors distinguish rurality × treatment strata as shown in the legend, and a black star marks the higher-performing model within each stratum. Large-metropolitan residents who received no treatment form the largest subgroup (37.0%). Abbreviations: FAIR-PLR = functionally adaptive interaction regularization–penalized logistic regression; PLR = penalized logistic regression; SU/MH = substance use or mental health; AUC-ROC = area under the receiver operating characteristic curve; TP% = true-positive percentage.

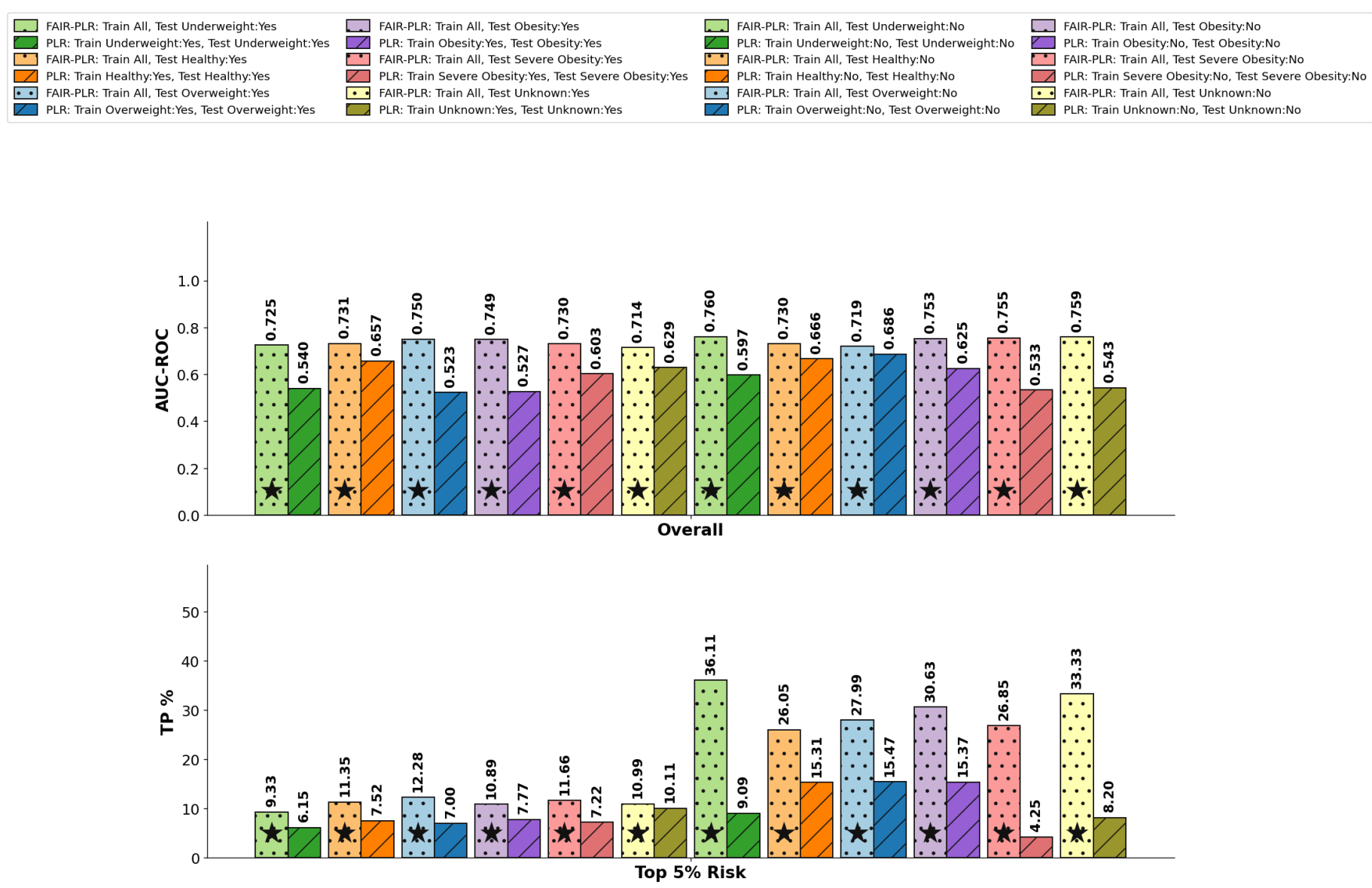

Supplementary Figure 7: **Performance of FAIR-PLR and separate PLR models across cross-sectional subgroups defined by body mass index (BMI) and serious psychological distress (SPD) status.** The top panel shows AUC-ROC and the bottom panel shows true-positive percentage (TP%) at the top 5% of predicted risk. Bars compare FAIR-PLR (dotted fill) and per-subgroup PLR (diagonal-hatch fill); colors distinguish BMI $\times$ SPD strata as shown in the legend, and a black star marks the higher-performing model within each stratum. Overweight individuals with SPD form the largest subgroup (27.1%). Abbreviations: FAIR-PLR = functionally adaptive interaction regularization–penalized logistic regression; PLR = penalized logistic regression; SPD = serious psychological distress; BMI = body mass index; AUC-ROC = area under the receiver operating characteristic curve; TP% = true-positive percentage.

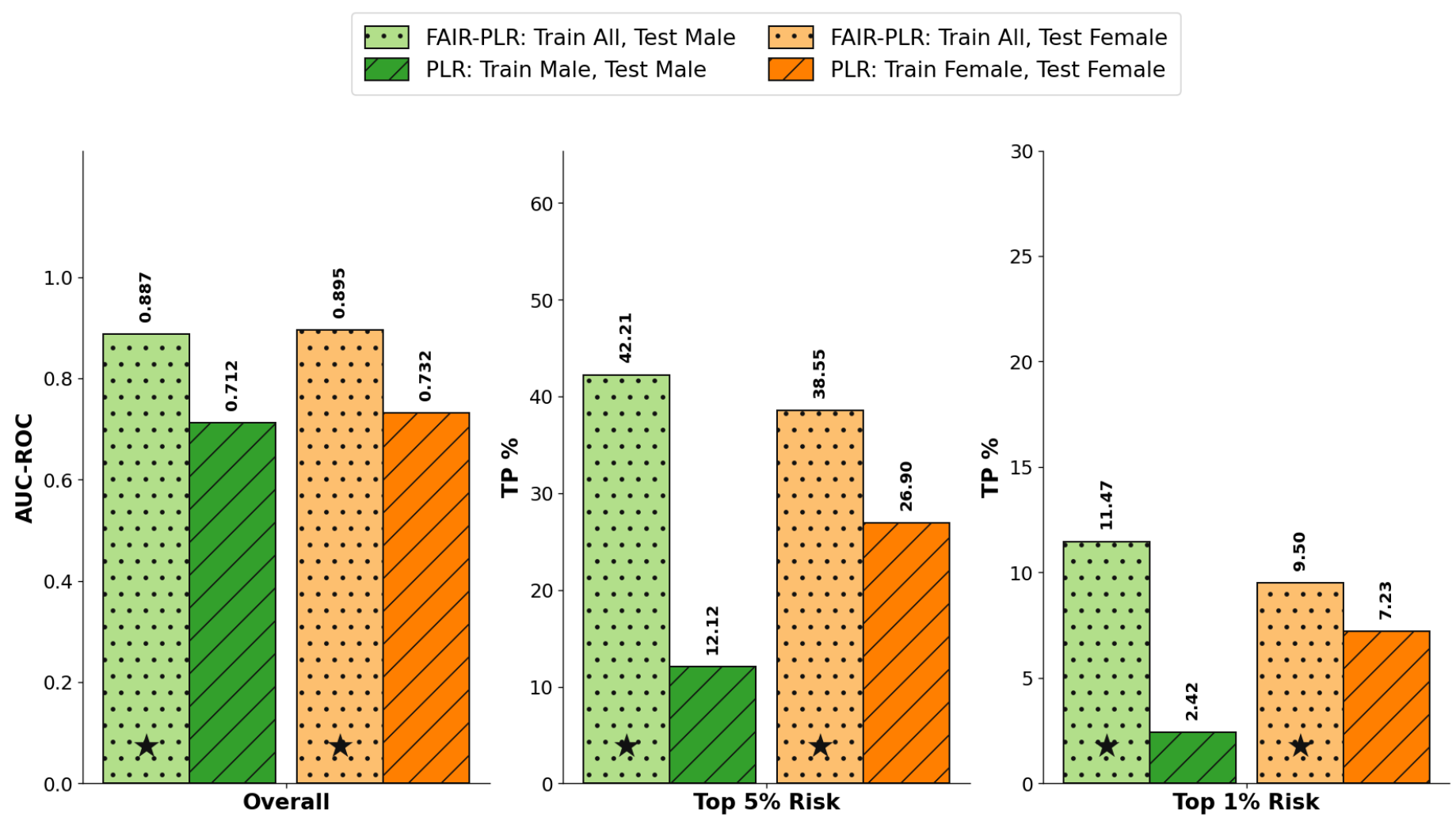

Supplementary Figure 8: **Performance of FAIR-PLR and separate PLR models across single sex subgroups.** Bars compare FAIR-PLR (dotted fill) trained on the full sample and evaluated within the indicated stratum against separate PLR (diagonal-hatch fill) trained and evaluated within that same stratum; colors distinguish strata as shown in the legend, and a black star marks the higher-performing model within each stratum. **a)** AUC-ROC. **b)** True-positive percentage at the top 5% of predicted risk. **c)** True-positive percentage at the top 1% of predicted risk. Females are the cohort majority (54.3%). Abbreviations: FAIR-PLR = functionally adaptive interaction regularization–penalized logistic regression; PLR = penalized logistic regression; AUC-ROC = area under the receiver operating characteristic curve; TP% = true-positive percentage.

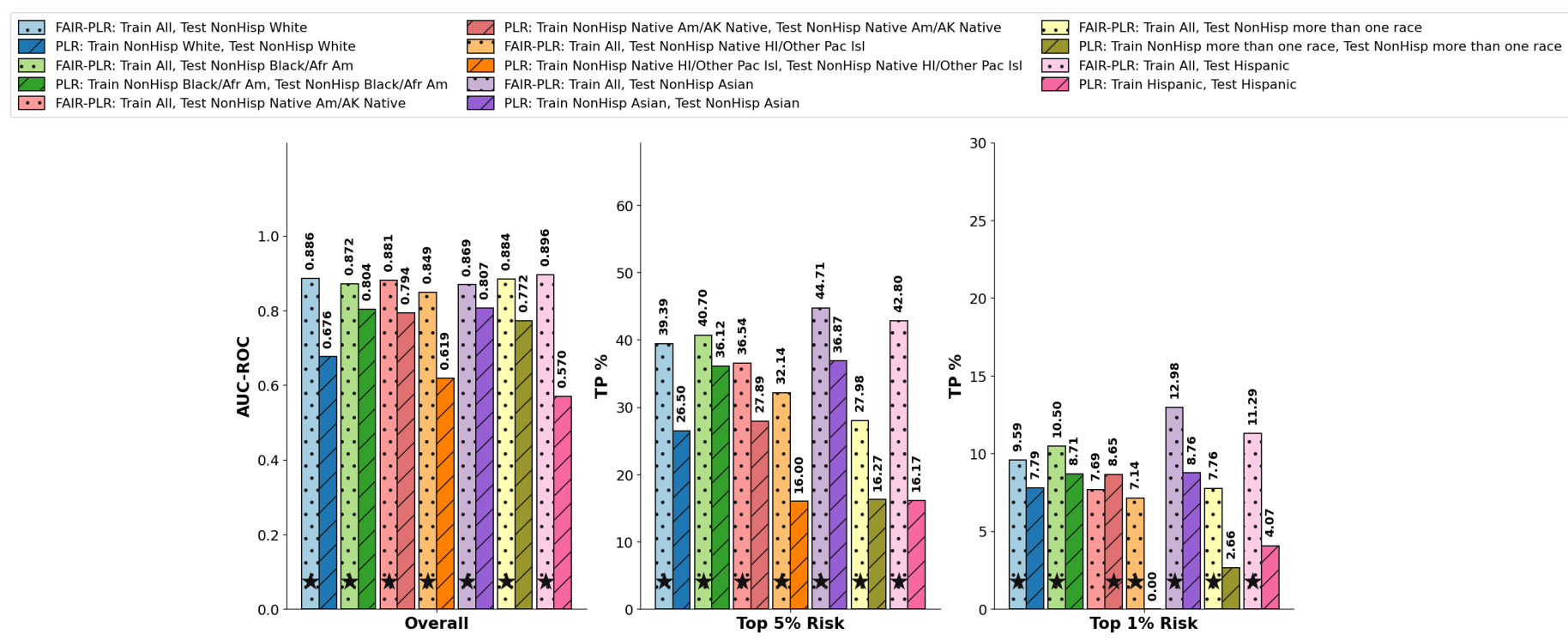

Supplementary Figure 9: **Performance of FAIR-PLR and separate PLR models across single race subgroups.** Bars compare FAIR-PLR (dotted fill) and per-subgroup PLR (diagonal-hatch fill); colors distinguish strata as shown in the legend, and a black star marks the higher-performing model within each stratum. **a)** AUC-ROC. **b)** True-positive percentage at the top 5% of predicted risk. **c)** True-positive percentage at the top 1% of predicted risk. Non-Hispanic White is the largest racial subgroup (60.9%). Abbreviations: FAIR-PLR = functionally adaptive interaction regularization–penalized logistic regression; PLR = penalized logistic regression; AUC-ROC = area under the receiver operating characteristic curve; TP% = true-positive percentage.

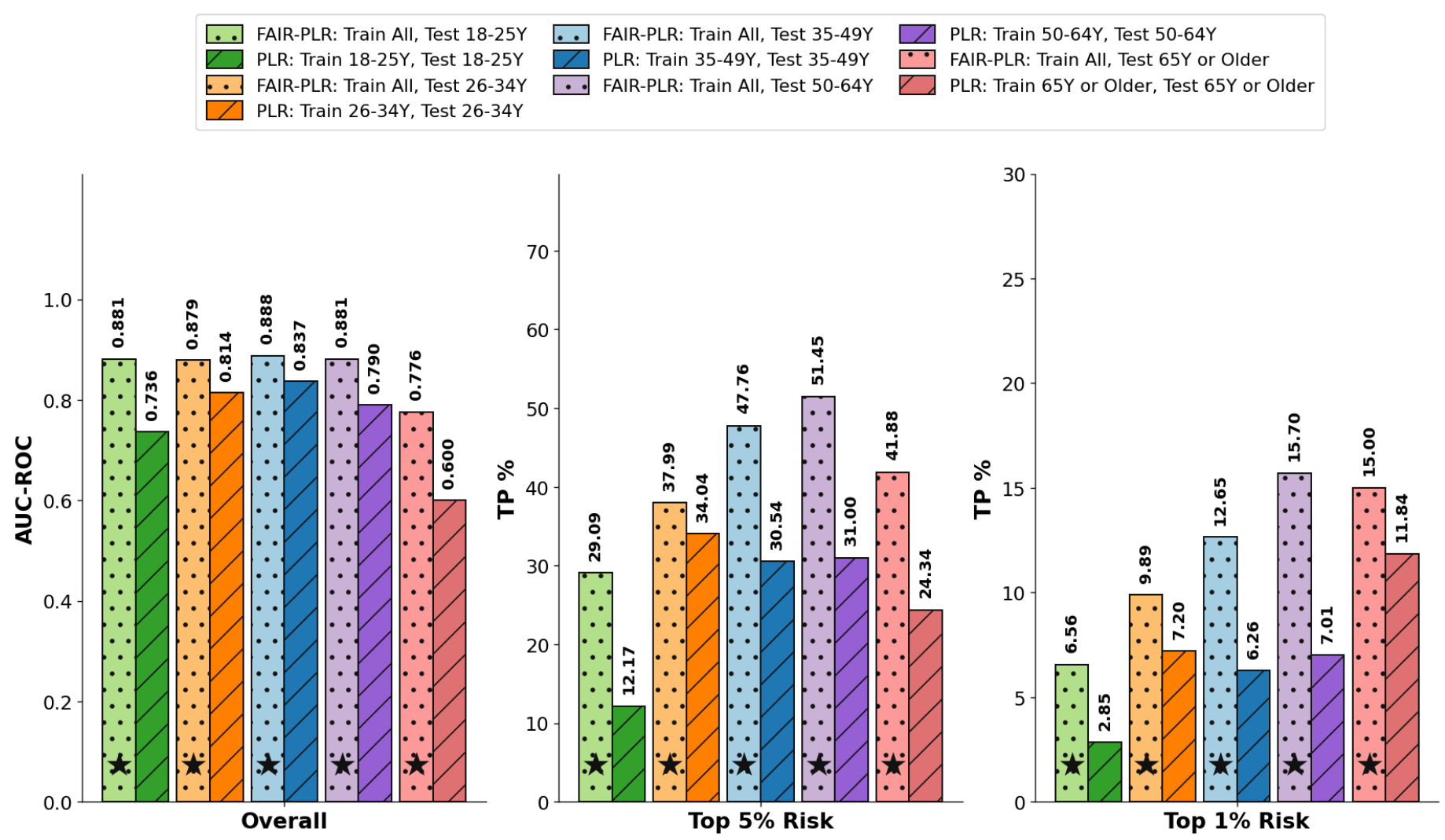

Supplementary Figure 10: **Performance of FAIR-PLR and separate PLR models across single age subgroups.** Bars compare FAIR-PLR (dotted fill) and per-subgroup PLR (diagonal-hatch fill); colors distinguish strata as shown in the legend, and a black star marks the higher-performing model within each stratum. **a)** AUC-ROC. **b)** True-positive percentage at the top 5% of predicted risk. **c)** True-positive percentage at the top 1% of predicted risk. Young adults (18–25) are the largest age subgroup (32.2%). Abbreviations: FAIR-PLR = functionally adaptive interaction regularization–penalized logistic regression; PLR = penalized logistic regression; AUC-ROC = area under the receiver operating characteristic curve; TP% = true-positive percentage.

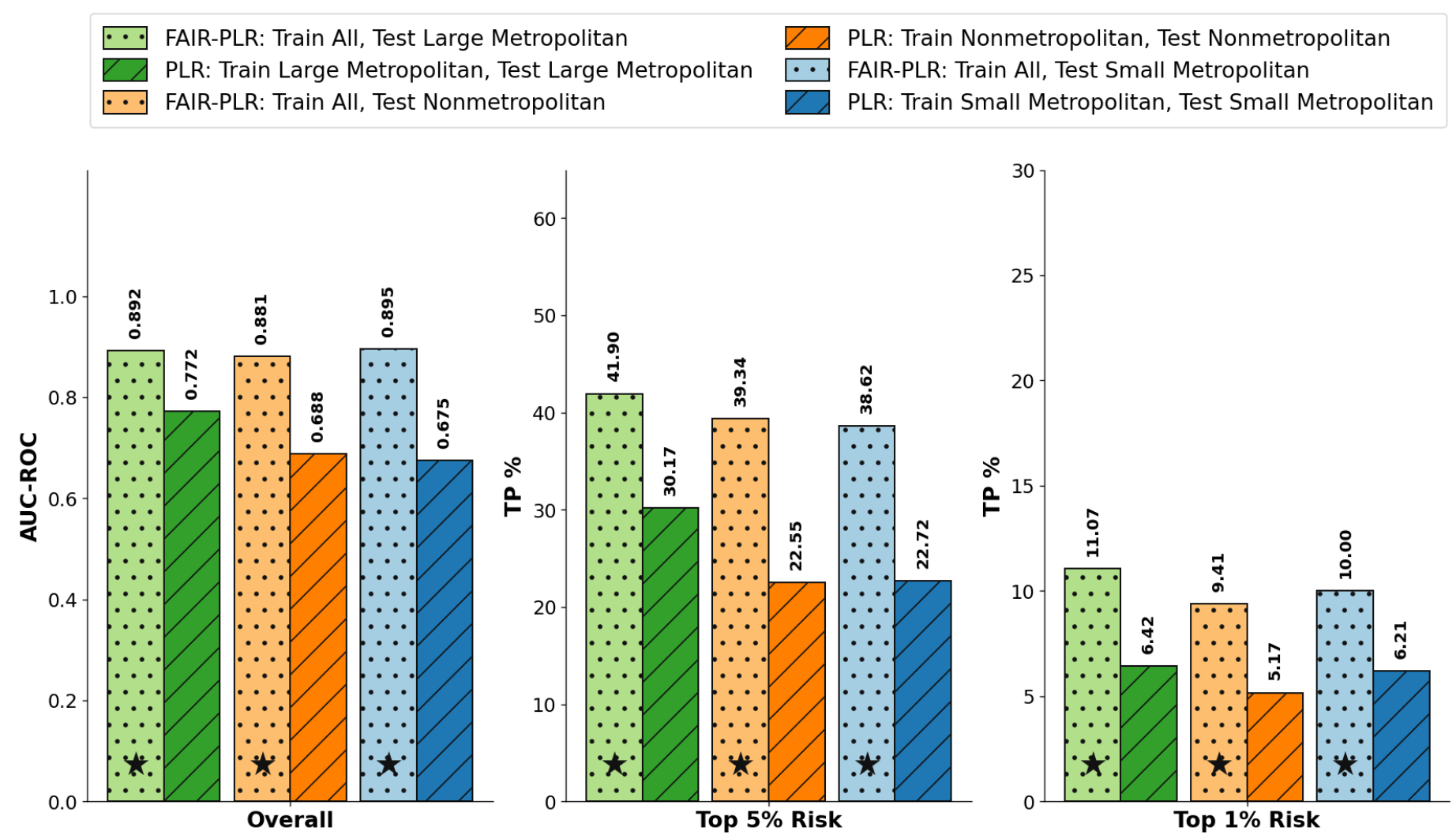

Supplementary Figure 11: **Performance of FAIR-PLR and separate PLR models across single rurality subgroups.** Bars compare FAIR-PLR (dotted fill) and per-subgroup PLR (diagonal-hatch fill); colors distinguish strata as shown in the legend, and a black star marks the higher-performing model within each stratum. **a)** AUC-ROC. **b)** True-positive percentage at the top 5% of predicted risk. **c)** True-positive percentage at the top 1% of predicted risk. Large metropolitan is the largest rurality subgroup (45.0%). Abbreviations: FAIR-PLR = functionally adaptive interaction regularization–penalized logistic regression; PLR = penalized logistic regression; AUC-ROC = area under the receiver operating characteristic curve; TP% = true-positive percentage.

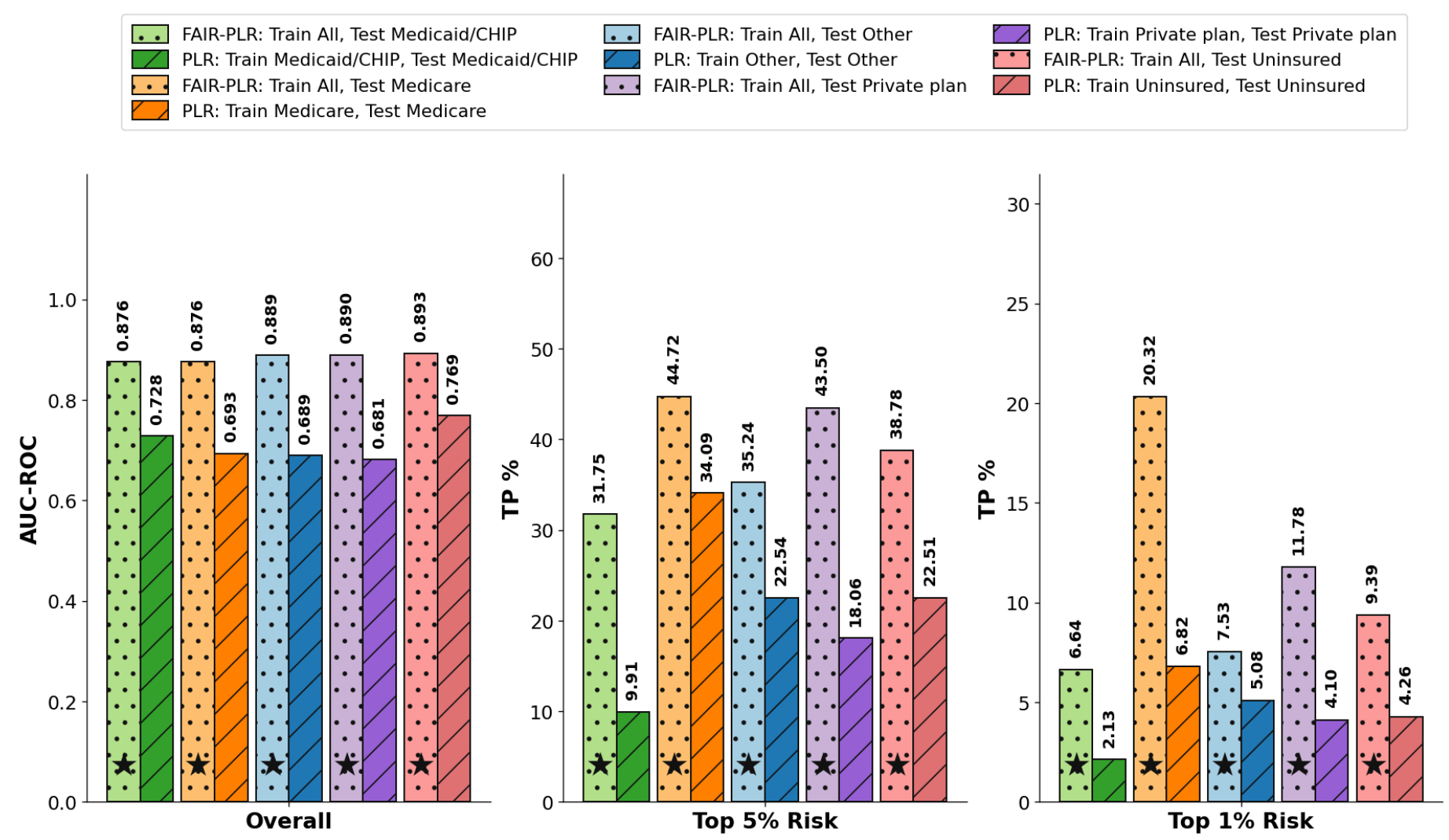

Supplementary Figure 12: **Performance of FAIR-PLR and separate PLR models across single health-insurance subgroups.** Bars compare FAIR-PLR (dotted fill) and per-subgroup PLR (diagonal-hatch fill); colors distinguish strata as shown in the legend, and a black star marks the higher-performing model within each stratum. **a)** AUC-ROC. **b)** True-positive percentage at the top 5% of predicted risk. **c)** True-positive percentage at the top 1% of predicted risk. Private-plan holders are the majority (62.5%). Abbreviations: FAIR-PLR = functionally adaptive interaction regularization–penalized logistic regression; PLR = penalized logistic regression; AUC-ROC = area under the receiver operating characteristic curve; TP% = true-positive percentage.

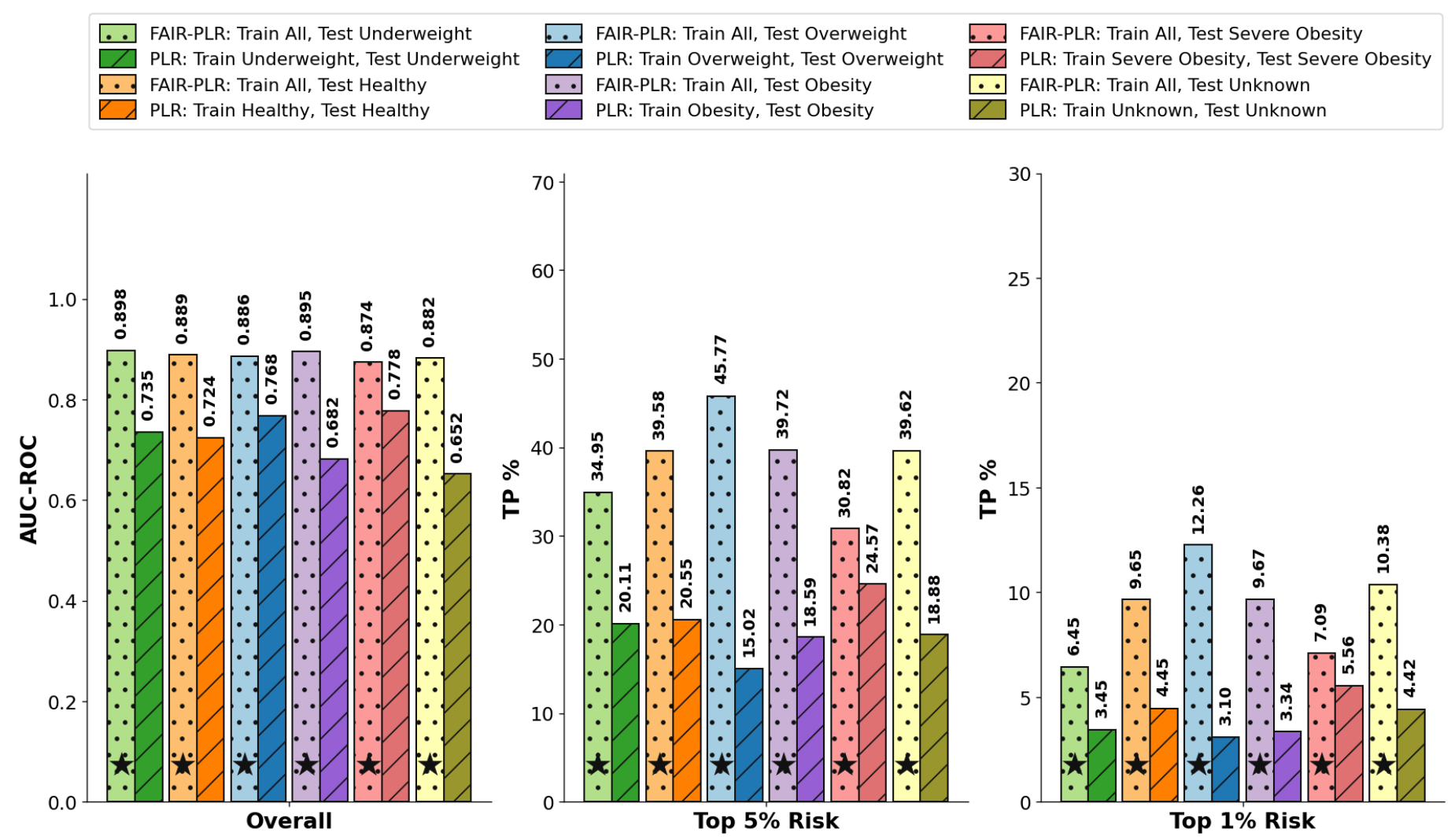

Supplementary Figure 13: **Performance of FAIR-PLR and separate PLR models across single body mass index (BMI) subgroups.** Bars compare FAIR-PLR (dotted fill) and per-subgroup PLR (diagonal-hatch fill); colors distinguish strata as shown in the legend, and a black star marks the higher-performing model within each stratum. **a)** AUC-ROC. **b)** True-positive percentage at the top 5% of predicted risk. **c)** True-positive percentage at the top 1% of predicted risk. Healthy BMI is the largest BMI subgroup (32.8%); FAIR-PLR enhances detection in the underweight and severe-obesity groups. Abbreviations: FAIR-PLR = functionally adaptive interaction regularization–penalized logistic regression; PLR = penalized logistic regression; BMI = body mass index; AUC-ROC = area under the receiver operating characteristic curve; TP% = true-positive percentage.

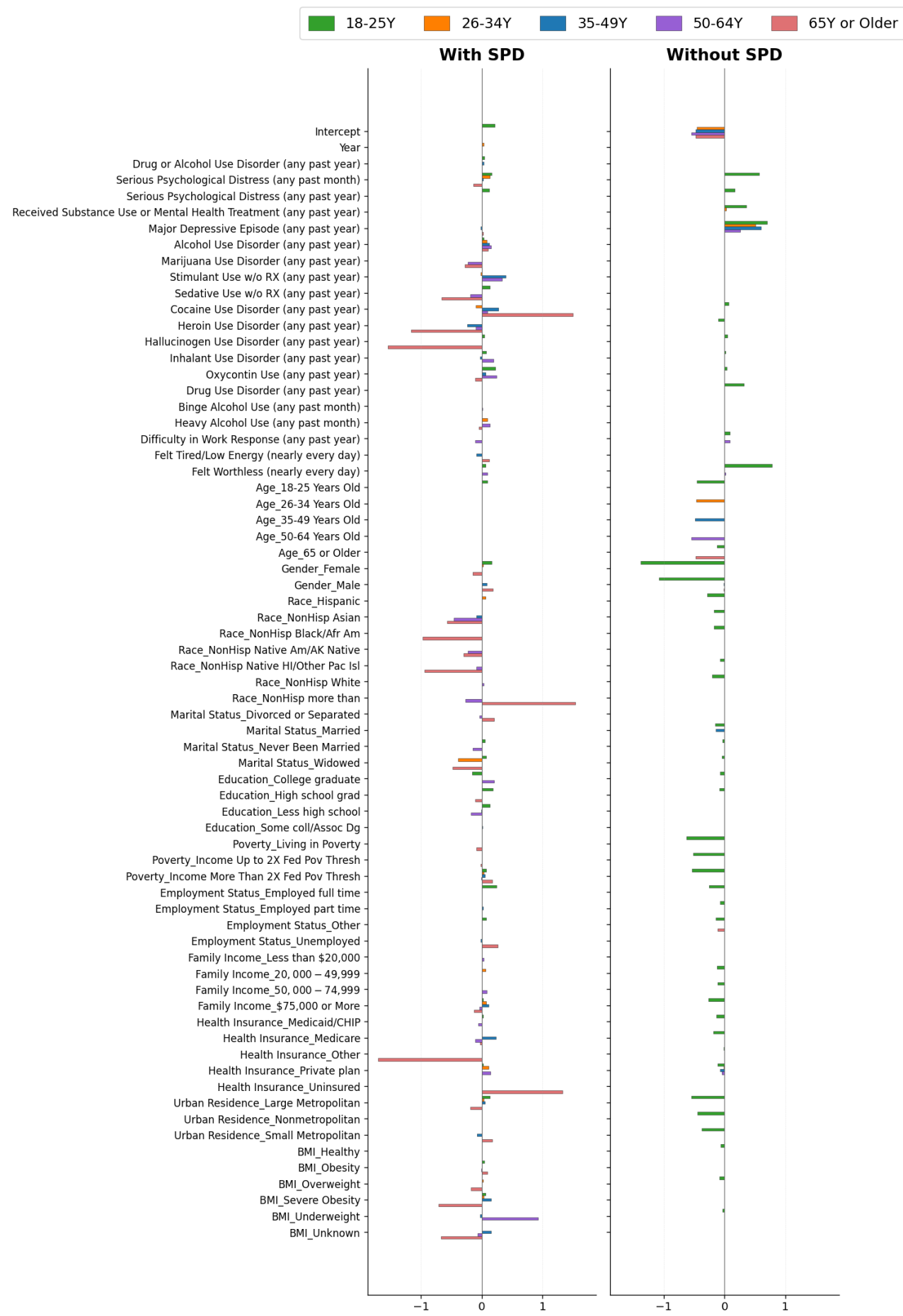

Supplementary Figure 14: **FAIR-PLR coefficients (log-odds) across Age $\times$ Serious Psychological Distress (SPD) subgroups.** Horizontal bars show adjusted feature effects; the vertical black line at zero marks no effect. Colors denote age categories (green = 18–25; orange = 26–34; blue = 35–49; purple = 50–64; pink = 65 or older). **a)** Coefficients for individuals with SPD. **b)** Coefficients for individuals without SPD. Abbreviations: FAIR-PLR = functionally adaptive interaction regularization–penalized logistic regression; SPD = serious psychological distress.

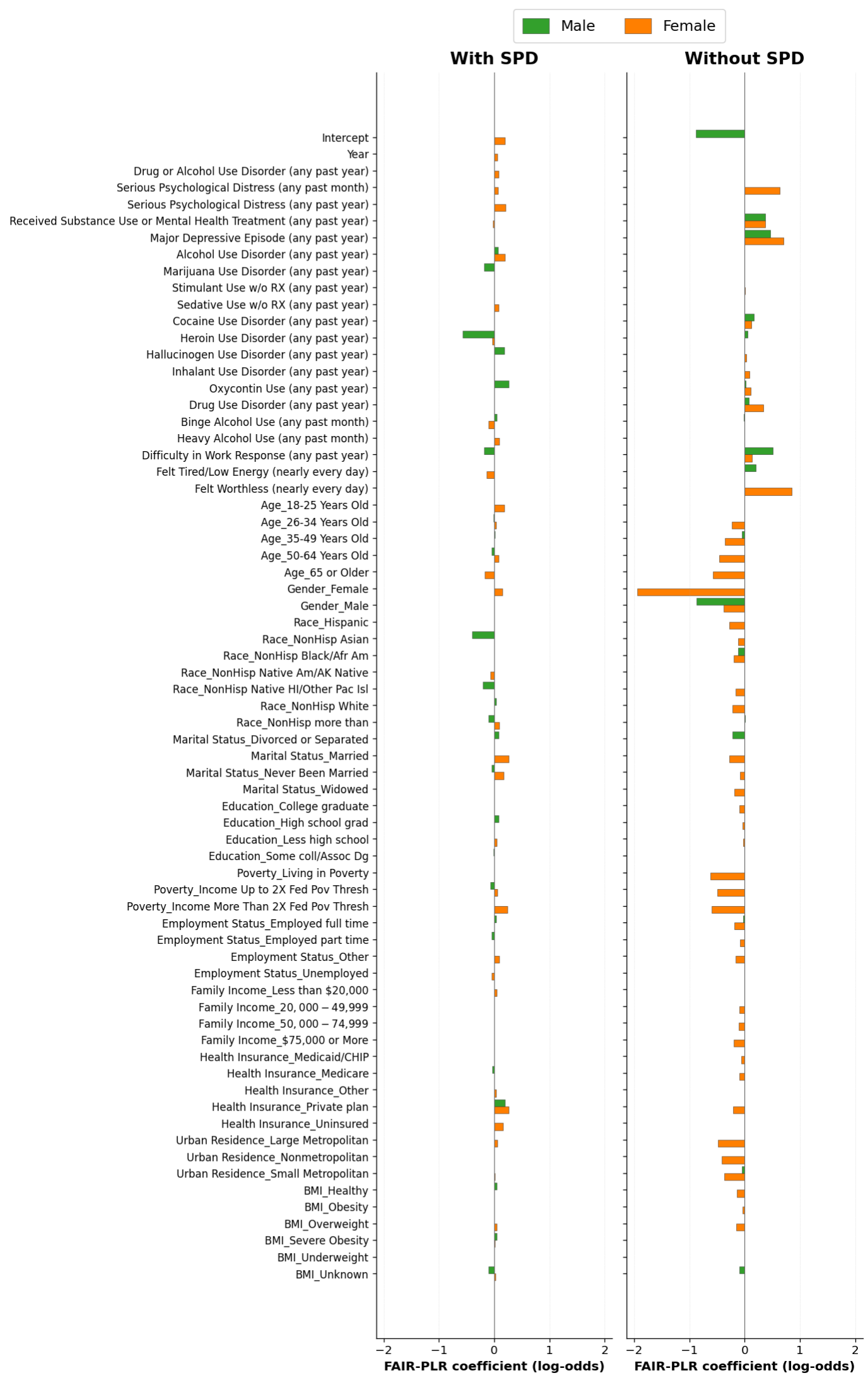

Supplementary Figure 15: **FAIR-PLR coefficients (log-odds) across Sex × Serious Psychological Distress (SPD) subgroups.** Horizontal bars show adjusted feature effects; the vertical black line at zero marks no effect. Colors denote sex (blue = male; pink = female). **a)** Coefficients for individuals with SPD. **b)** Coefficients for individuals without SPD. Abbreviations: FAIR-PLR = functionally adaptive interaction regularization–penalized logistic regression; SPD = serious psychological distress.

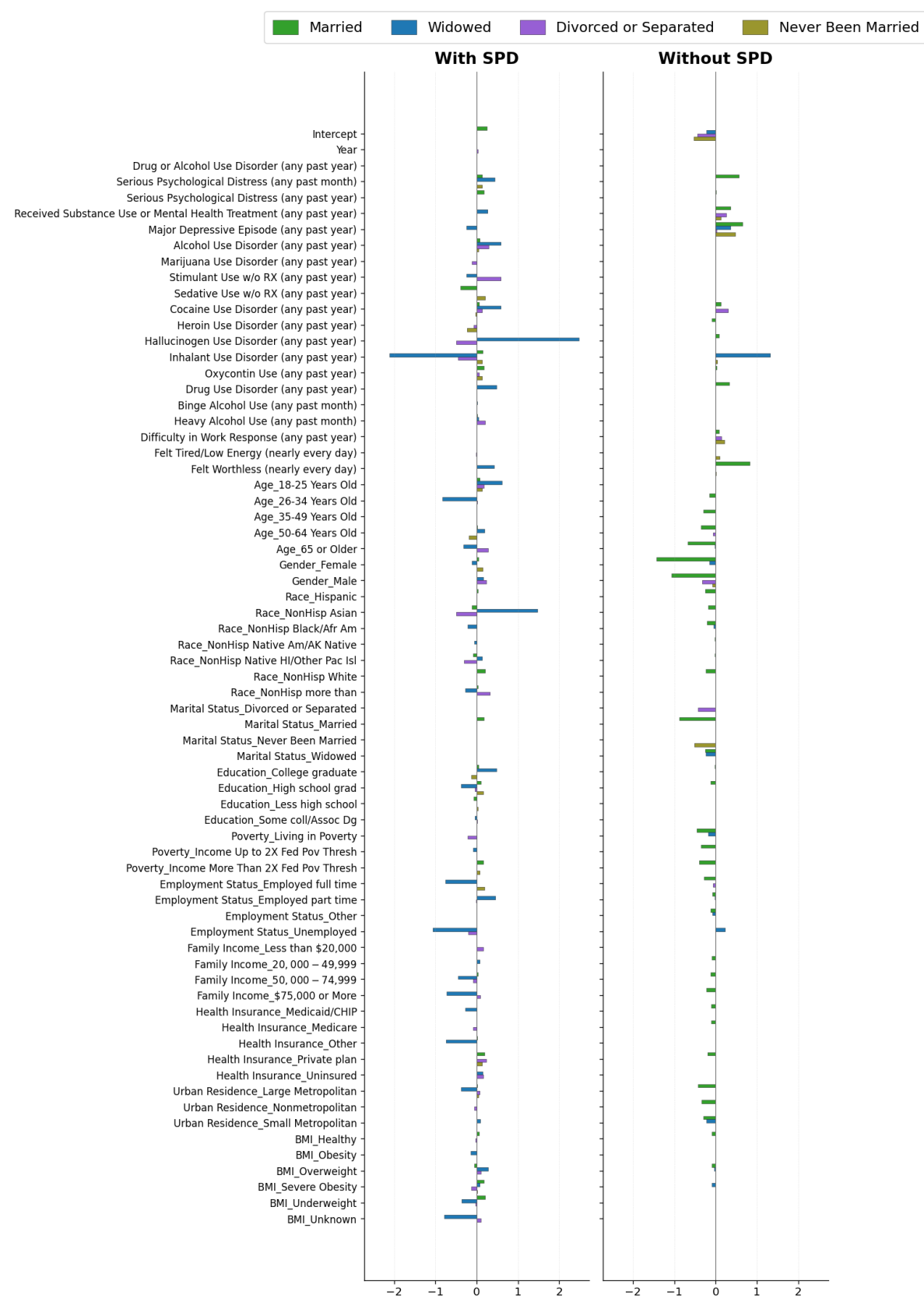

Supplementary Figure 16: **FAIR-PLR coefficients (log-odds) across Marital Status × Serious Psychological Distress (SPD) subgroups.** Horizontal bars show adjusted feature effects; the vertical black line at zero marks no effect. Colors denote marital status (green = Married; blue = Widowed; purple = Divorced or Separated; olive = Never Been Married). **a)** Coefficients for individuals with SPD. **b)** Coefficients for individuals without SPD. Abbreviations: FAIR-PLR = functionally adaptive interaction regularization–penalized logistic regression; SPD = serious psychological distress.

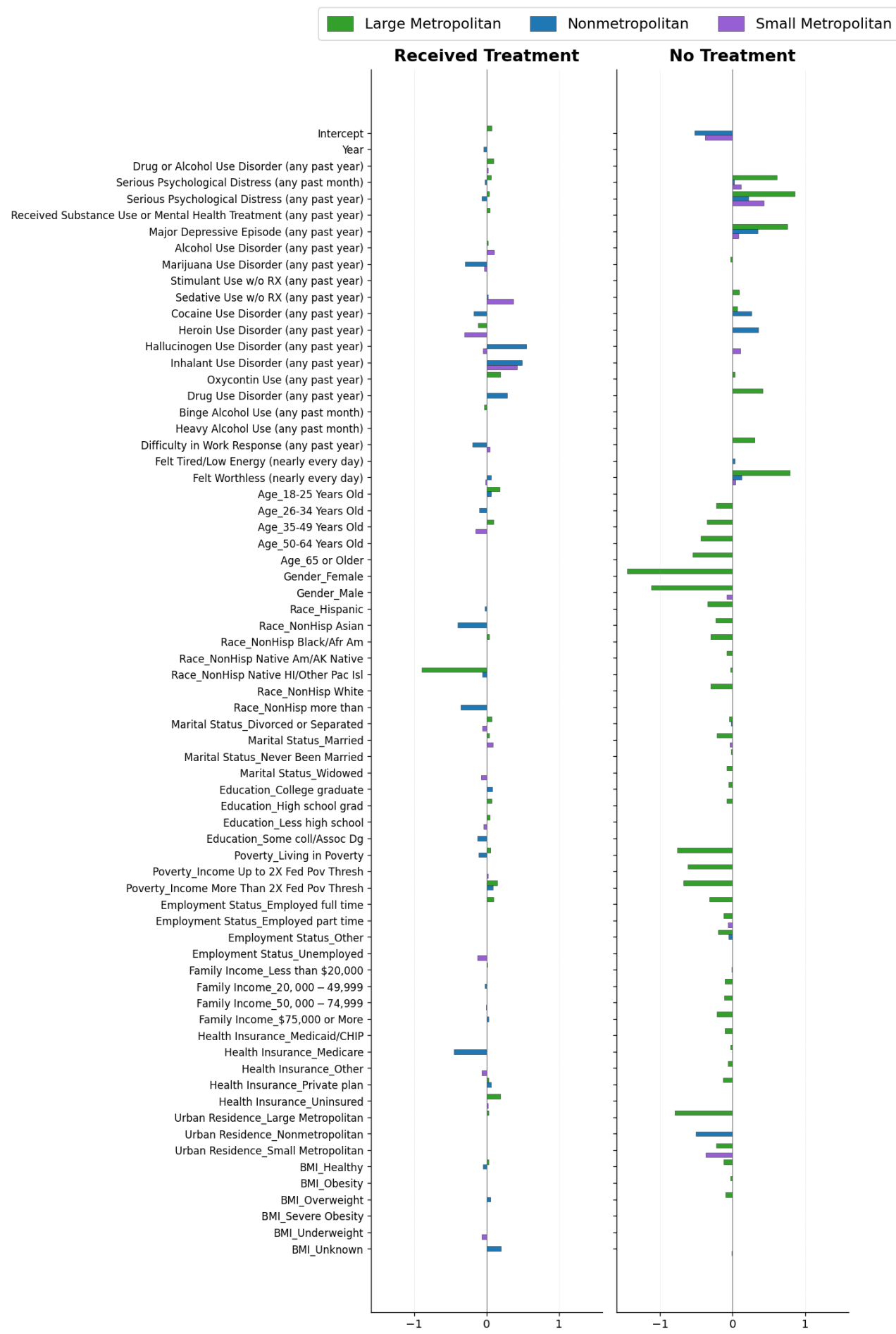

Supplementary Figure 17: **FAIR-PLR coefficients (log-odds) across Rurality $\times$ Treatment subgroups.** Horizontal bars show adjusted feature effects; the vertical black line at zero marks no effect. Colors denote rural-residence categories as shown in the legend. **a)** Coefficients for individuals who received substance-use or mental-health treatment in the past year. **b)** Coefficients for individuals who did not. Abbreviations: FAIR-PLR = functionally adaptive interaction regularization–penalized logistic regression; SU/MH = substance use or mental health.

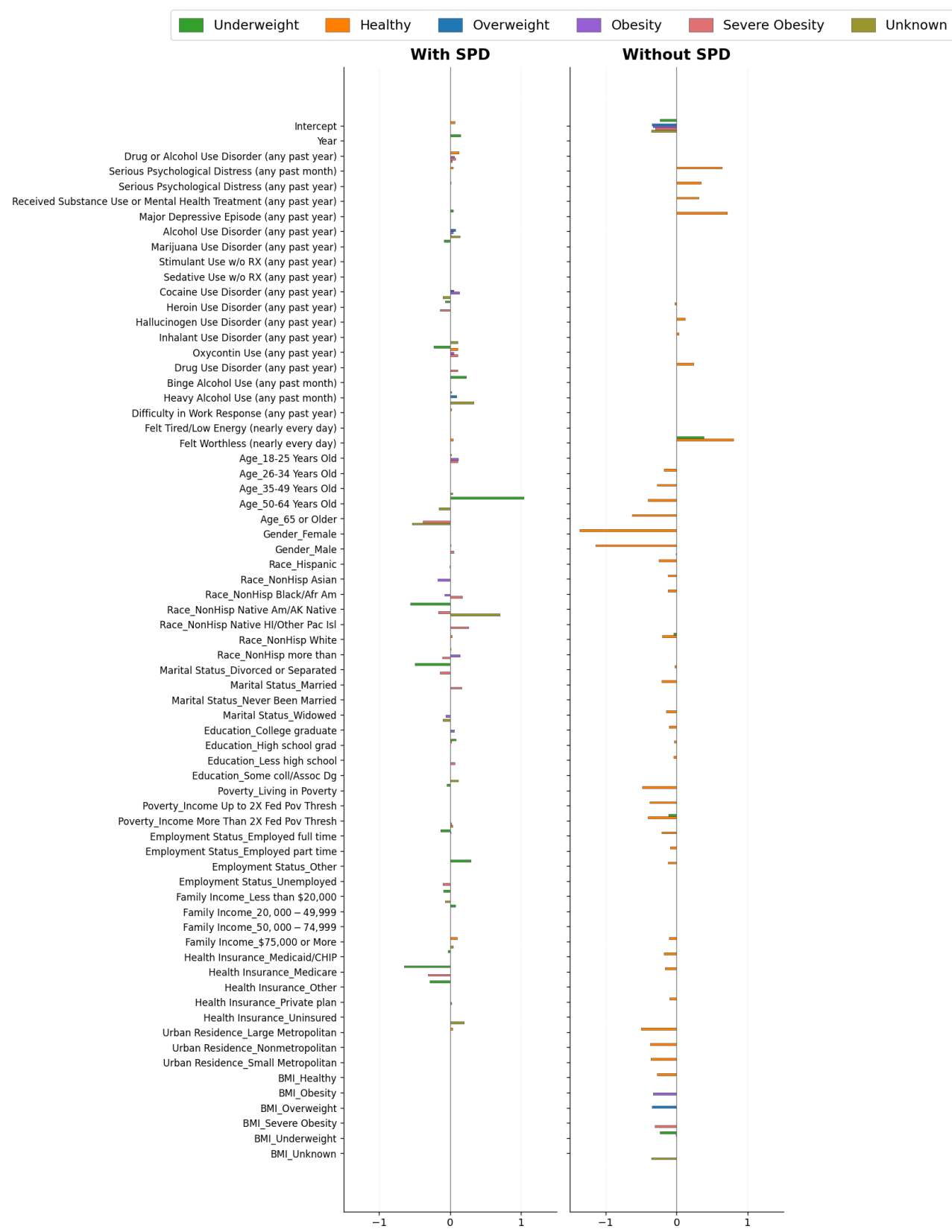

Supplementary Figure 18: **FAIR-PLR coefficients (log-odds) across Body Mass Index (BMI) × Serious Psychological Distress (SPD) subgroups.** Horizontal bars show adjusted feature effects; the vertical black line at zero marks no effect. Colors denote BMI categories as shown in the legend. **a)** Coefficients for individuals with SPD. **b)** Coefficients for individuals without SPD. Abbreviations: FAIR-PLR = functionally adaptive interaction regularization–penalized logistic regression; SPD = serious psychological distress; BMI = body mass index.

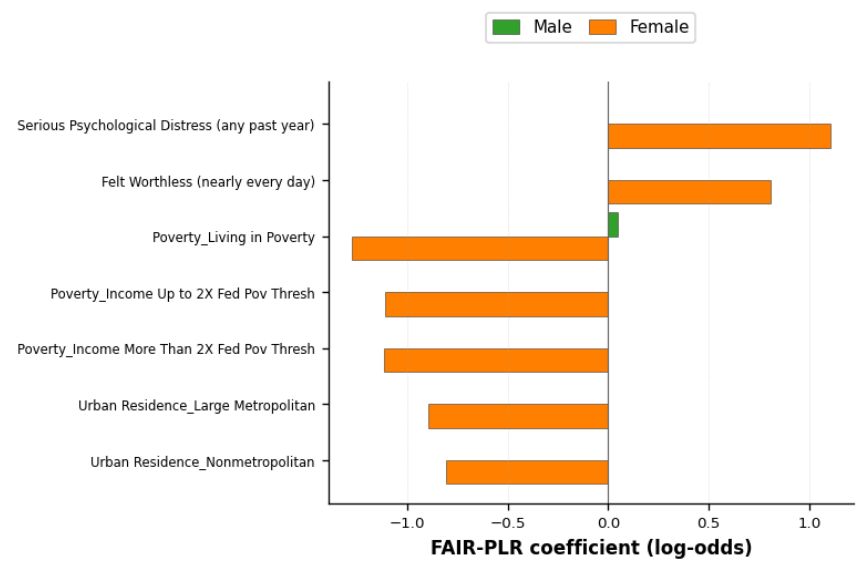

(a) Sex-Fair Model

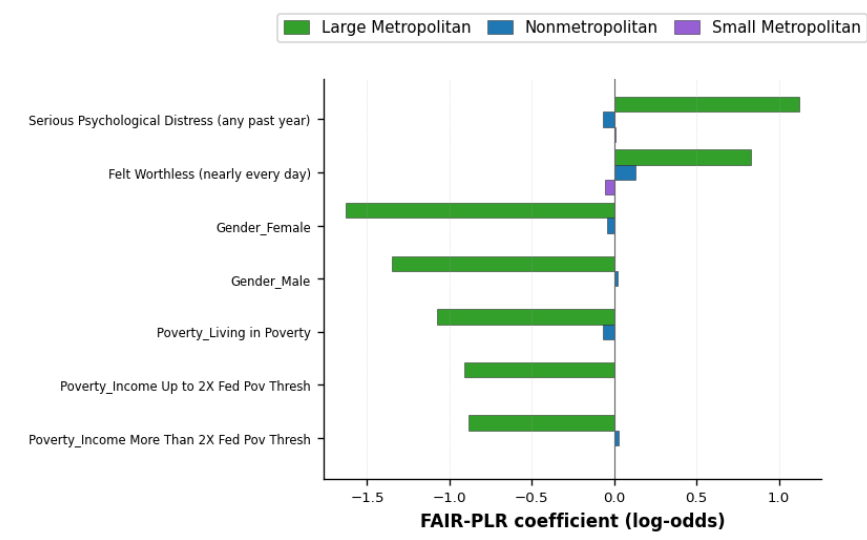

(b) Rurality-Fair Model

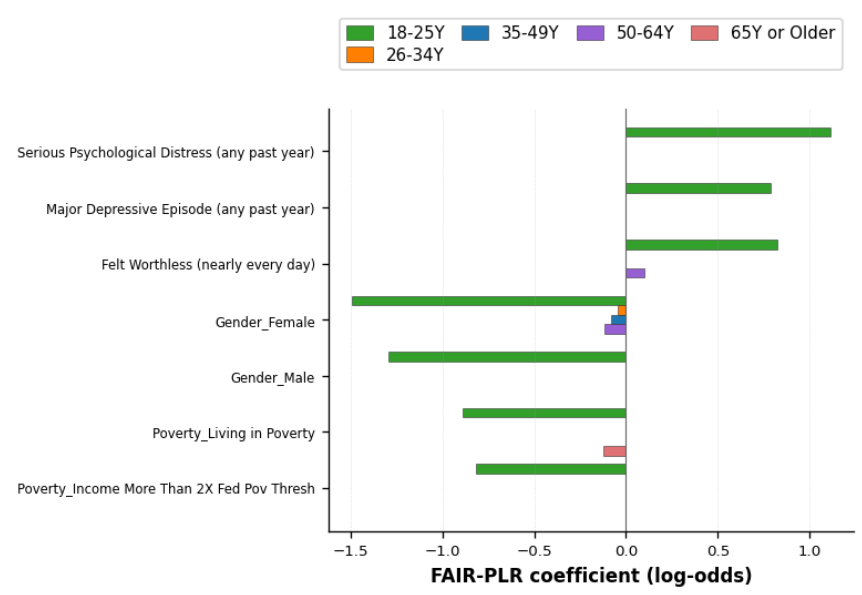

(c) Age-Fair Model

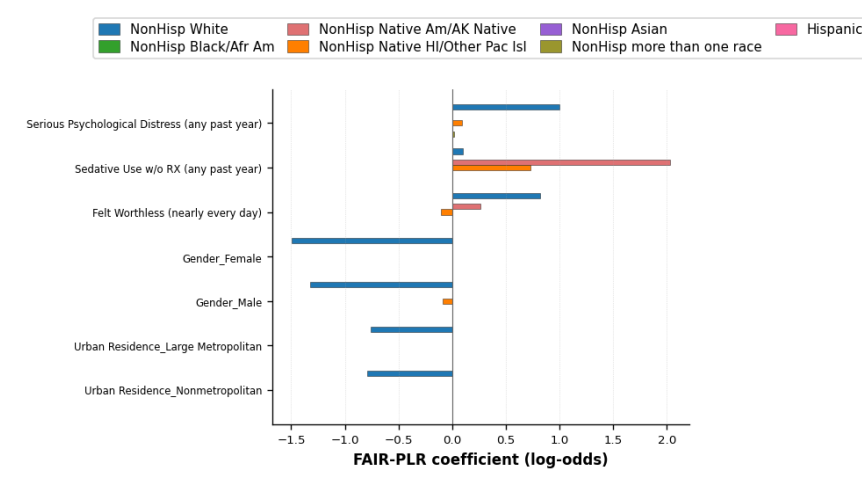

(d) Race-Fair Model

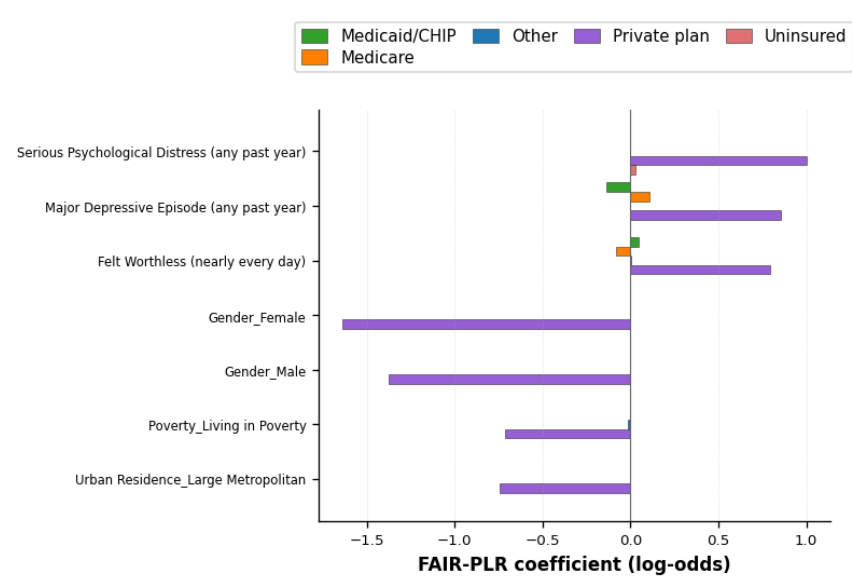

(e) Insurance-Fair Model

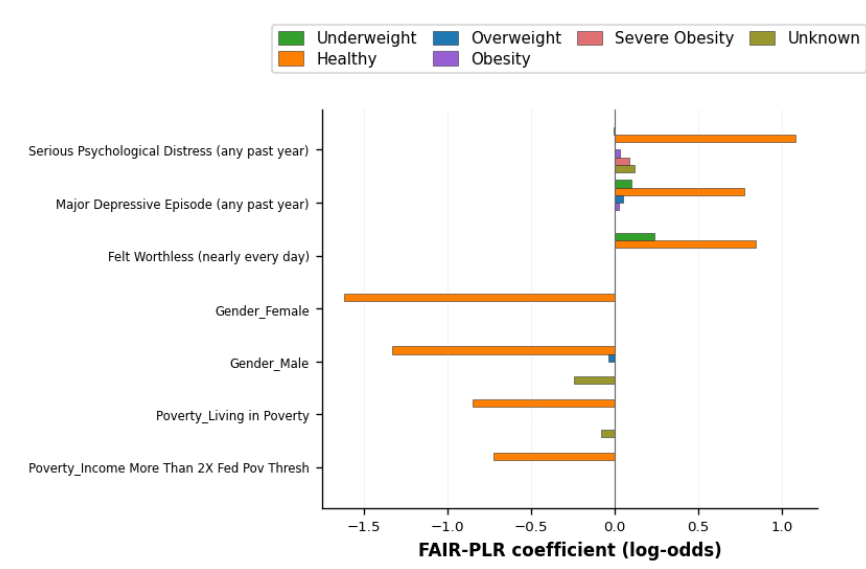

(f) BMI-Fair Model

Supplementary Figure 19: **Subgroup-specific FAIR-PLR coefficients showing the most influential predictors within each single-subgroup model.** Horizontal bars give coefficient values (log-odds); positive bars indicate higher predicted suicide risk and negative bars indicate lower predicted risk, and colors distinguish strata within each panel as shown in the panel legends. a) Sex-Fair model. b) Rurality-Fair model. c) Age-Fair model. d) Race-Fair model. e) Health Insurance-Fair model. f) BMI-Fair model. Abbreviations: FAIR-PLR = functionally adaptive interaction regularization–penalized logistic regression; BMI = body mass index.
